# Defect Migration in Supercrystalline Nanocomposites

**DOI:** 10.1021/acsnano.5c16138

**Published:** 2025-12-17

**Authors:** Dmitry Lapkin, Cong Yan, Emre Gürsoy, Hadas Sternlicht, Alexander Plunkett, Büsra Bor, Young Yong Kim, Dameli Assalauova, Fabian Westermeier, Michael Sprung, Tobias Krekeler, Surya S. Rout, Martin Ritter, Satishkumar Kulkarni, Thomas F. Keller, Gerold A. Schneider, Gregor B. Vonbun-Feldbauer, Robert H. Meissner, Andreas Stierle, Ivan A. Vartanyants, Diletta Giuntini

**Affiliations:** † 28332 Deutsches Elektronen-Synchrotron DESY, Hamburg 22607, Germany; ‡ Department of Mechanical Engineering, 3169 Eindhoven University of Technology, Eindhoven 5612 AP, Netherlands; § Institute for Interface Physics and Engineering, 38987 Hamburg University of Technology, Hamburg 21073, Germany; ∥ Department of Materials Science and Engineering, The Pennsylvania State University, University Park, Pennsylvania 16802, United States; ⊥ Institute of Advanced Ceramics, Hamburg University of Technology, Hamburg 21073, Germany; # Electron Microscopy Unit, Hamburg University of Technology, Hamburg 21073, Germany; ∇ Centre for X-ray and Nano Science, Deutsches Elektronen-Synchrotron DESY, Hamburg 22607, Germany; ○ Department of Physics, University of Hamburg, Hamburg 22607, Germany; ◆ Institute of Surface Science, Helmholtz-Zentrum Hereon, Geesthacht 21502, Germany

**Keywords:** supercrystals, nanocomposites, defects, self-assembly, annealing

## Abstract

Supercrystalline nanocomposites (SCNCs) are nanostructured hybrid materials with a variety of unique functional properties. Given their periodically arranged building blocks, they also offer interesting parallels with crystalline materials. They can be processed in multiple forms and at different scales, and cross-linking their organic ligands via heat treatment leads to a boost of their mechanical properties. This study shows, via X-ray and in situ scanning transmission electron microscopy (STEM) analyses, how each of these processing steps plays a distinct role in the generation, migration, interaction, and healing of supercrystalline defects. Pressing of SCNCs into bulk pellets leads to a distortion of the otherwise *fcc* superlattice, while emulsion-templated self-assembly yields supraparticles (SPs) with stacking faults and size-dependent symmetries. Heat treatment at the same temperatures as those applied for the organic cross-linking has significant effects on planar defects. Stacking faults migrate and get healed, as also confirmed via molecular dynamics simulations, and intersupercrystalline “grain” boundaries migrate via anisotropic motion of disconnections. These rearrangements of defects at the supercrystalline scale (tens of nanometers) in nanocomposites with high mechanical properties (compressive strength of 100–500 MPa) provide insights into the formation and evolution of ordered assemblies of functionalized nanoparticles.

## Graphical abstract

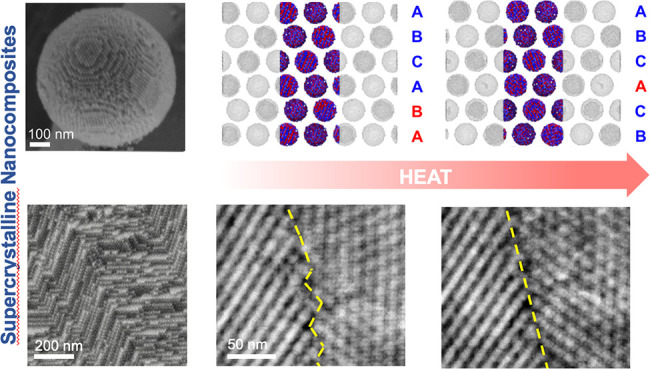

Supercrystalline nanocomposites (SCNCs) are new remarkable materials consisting of self-assembled inorganic nanoparticles (NPs) that are surface-functionalized with organic ligands.^1–6^ This combination of nanosized building blocks and their long-range ordered arrangement, analogous to that of atoms in crystals, is emerging as a powerful material design strategy. By tailoring composition, NP size, arrangement, and spacings, emergent collective properties can be fostered, with promising applications in, e.g., the catalysis, energy, optoelectronics, and magnetic materials fields.^1,4,7–10^ Beyond this spectrum of potential applications, SCNCs are also studied in light of available knowledge on conventional crystalline materials.^11^ Crystalline structures, phases, and defects have long been investigated with systems of periodically arranged building blocks at larger scales—starting with bubble rafts,^12^ through colloidal crystals of unfunctionalized microparticles,^13^ all the way to the recent progress made with bimodal distributions of DNA-functionalized NPs.^14^

Two main issues, however, still stand in the way of SCNCs’ implementation into devices: controlling their assembly into scales beyond 2D materials and microsized domains, and increasing their mechanical robustness.^5,15^ On the former aspect, progress is being made by self-assembly controlled via targeted ligand interactions;^16^ conducting self-assembly in macro-scale geometries followed by a pressing processing step;^17,18^ or via hierarchical designs.^19,20^ On the mechanical properties side, heat treatment and organic cross-linking have been proven to be very effective means toward stabilizing, strengthening, stiffening, hardening, and even toughening SCNCs.^5,17,21–29^

As in any material system, all of these aspects are strongly influenced by defects. Defects control the mechanical and functional performance of a material, and they are thus a feature to tune in the development of programmable materials.^30^ Research on how processing affects defects in SCNCs, and how defects in turn affect material properties and performance, is, however, still in its infancy. Most studies have focused on the occurrence of imperfections during the assembly of colloidal crystals of unfunctionalized microparticles.^31–40^ A few have considered mechanically induced defects.^41^ The important aspects of defect mobility, migration, interaction, and healing have been addressed so far mainly for single-component colloidal crystals, i.e., periodic arrays of unfunctionalized microparticles.^42^ Most of these studies focus on defect migration during self-assembly,^42^ and only a few on the postassembly stages.^43–51^ In all of these cases, the colloidal crystals are in a soft matter state, which is significantly different with respect to the strong SCNCs.^5^

For SCNCs, most investigations on superlattice defects focus on their formation during self-assembly in thin films^52,53^ or microsized single supercrystals.^54,55^ Mechanically induced defects are just starting to be analyzed, while their migration remains unexplored.^24,56^ Annealing (heat-treating) SCNCs, on the other hand, is starting to provide meaningful insights on the occurrence of strain or phase transitions in superlattices.^57,58^ So far these transitions have been addressed via superlattice “melting” in temperature ranges well below 100 °C, and mainly for DNA-based NP superlattices.^59–62^ SCNCs, however, are processable in a broad spectrum of compositions and in states that go well beyond soft matter. Once the organic ligands are cross-linked, the high values of strength, hardness, and elastic modulus that they reach fully qualify these materials as hard composites.^5,21,25^ This kind of ultrastrong SCNC is processed in two- or three-step routines involving self-assembly and a heat treatment. Each of these steps has a chance to induce supercrystalline defects or potentially allow their migration and healing.

This work explores these aspects for inorganic–organic SCNCs via a combination of X-ray scattering and in situ heating scanning transmission electron microscopy (STEM). Performing scattering experiments while rotating the sample by a small angular increment in a small-angle X-ray scattering (SAXS) regime enables the determination of the full 3D reciprocal space map of the sample. Angular X-ray Cross-Correlation Analysis (AXCCA) of the 3D intensity distribution enables the identification not only of the average structure of the supercrystalline sample but also of potential defects.^63–65^ STEM, on the other hand, allows localized structural characterization at higher magnifications.^66^ We show here that, while all SCNCs feature predominantly face-centered cubic (*fcc*) superlattice arrangements, different types of lattice distortions and defects are detected in differently processed SCNCs. Pressing bulk SCNC pellets leads to a “stretching” of the *fcc* superlattice into a slightly distorted triclinic one, while spherical supercrystalline supraparticles (SPs) show the presence of random hexagonal close-packed (*r-hcp*)^67,68^ motifs within a prevalently *fcc* structure. Remarkably, heating the SCNCs at mild temperatures (up to 350 °C)—such as those inducing organic cross-linking—leads to the migration and healing of defects, as revealed for both superlattice stacking faults and intersupercrystalline (“grain”) boundaries.

## Results and Discussion

### SCNCs Nanostructure and Mechanical Properties

The supercrystalline structure is obtained via self-assembly. To obtain macroscopic materials, self-assembly is conducted with two strategies: (1) solvent destabilization with the initial suspension of functionalized NPs in a die-punch assembly; (2) emulsion-templated self-assembly.^21,69^ Method (1) yields bulk, millimeter-sized samples, which are subsequently pressed uniaxially to shape the material into pellets. This self-assembly method results in poly-supercrystalline materials, i.e., containing multiple superlattice domains with varying orientations, analogous to grains in polycrystalline materials. Method (2) yields a distribution of μm-sized supercrystalline spheres, i.e., supraparticles (SPs),^69,70^ which can be used as building blocks for hierarchical bulk materials.^71^ In all cases, the material building blocks are quasi-spherical (truncated cuboctahedral) iron oxide (magnetite, Fe_3_O_4_) NPs, with a radius of 7.4 ± 0.8 nm, surface-functionalized with oleic acid (Fraunhofer CAN GmbH, Hamburg, Germany). For the following analysis, samples with characteristic sizes in the μm range are considered to assess no more than two supercrystalline domains at a time. From the bulk pellets, micropillars and lamellae are obtained via focused ion beam (FIB) milling, the former with a square cross-section to facilitate the subsequent X-ray analysis, and the latter for the STEM analysis. All details on SCNC processing are reported in previous publications^5,21,69,72^ and briefly summarized in the Methods and (Supporting Information SI), section 1.

We therefore study two types of SCNCs: micropillars from bulk pellets (named “Pillars” in the following section) and individual supraparticles (“SPs”). Figure 1 shows SEM micrographs of the two types of SCNCs: Pillars with varying superlattice orientations are shown in Figure 1a–c and SPs in Figure 1d–f. As the fracture surfaces of Figure 1b,c show, in the bulk pellets, surface ledges (surface steps) and terraces can be noted along specific planes due to the supercrystalline anisotropy. The same applies to the surface of SPs.^73^ The presence of anisotropic ledges and terraces agrees with those previously detected at interfaces in conventional polycrystalline systems,^74–77^ even though at a markedly larger length scale. In SPs, the superlattice orientation must be accommodated within the spherical shape. In line with previous reports, a finite number of particles (above 100) organizes into an icosahedral symmetry with either Mackay or anti-Mackay structures (Figure 1e),^38,78^ while for particle numbers above 10^6^, the structure transitions into single *fcc* domains (Figure 1f).^37,38^

**Figure 1:**
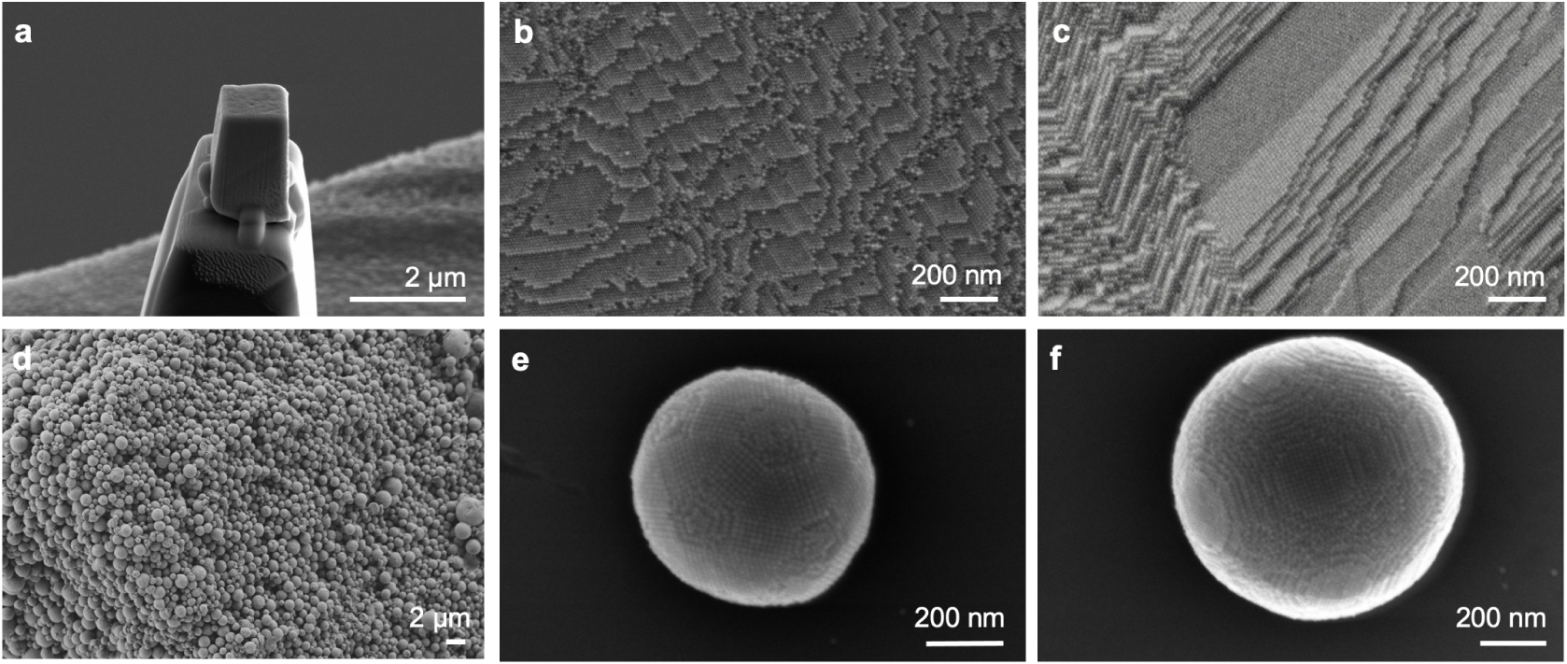
Nanostructure of the supercrystalline nanocomposites (SCNCs). (a–c) Micropillar (“Pillar”) from bulk material with representative nanostructures. (a) Pillar fixed on top of the pin used for the 3D X-ray analysis. (b,c) Secondary electron (SE) images of the supercrystalline nanostructure from fracture surfaces of bulk SCNCs, with the organically functionalized iron oxide NPs organized in periodic arrangements with multiple orientations, representative of the poly-supercrystalline character of the bulk samples. (d–f) SE images of supraparticles (SPs) were obtained via emulsion-templated self-assembly. (d) Overview of the gram-scale production of iron oxide-oleic acid SPs.^69^ (e,f) Single SPs with evidence of (e) anti-Mackay superlattice in SP 2 and (f) single *fcc* arrangement of the functionalized NPs in SP 3.

A key processing step in both cases is the heat treatment-induced cross-linking of the organic phase. In this system, cross-linking can be induced via heat treatment in a broad spectrum of temperatures and atmospheres.^25^ In this study, we focus on heating for 18 min at 325 °C in a N_2_ atmosphere, with a heating ramp of 1 °C·min^‑1^, which is one of the most efficient and well-established routines.^21^ Cross-linking of the organic phase leads to the transition from a material held together mainly by van der Waals interactions to one in which the NPs are interconnected through a network of strong covalent bonds. This results in a significant increase in elastic modulus (up to ∼65 GPa), hardness (beyond 4 GPa), and strength (>500 MPa in bending and >1 GPa in compression).^5,21^ It should nevertheless be mentioned that even before the heat treatment, the SCNCs, both in bulk and in SP forms, already show remarkably high mechanical properties (elastic modulus ∼15 GPa, hardness >0.5 GPa, compressive and bending strength >100 MPa).^17^ Other types of SCNCs typically feature, instead, elastic modulus in the 0.1–10 GPa range and hardness ∼10–200 MPa.^79,80^

Samples were measured by means of X-ray scattering, with a μm-sized focused synchrotron X-ray beam, before and after heat treatment. Three Pillars and 3 SPs were analyzed. Pillars 1 and 2 are tested once heat-treated (“HT”), while Pillar 3 is tested both before and after heat treatment. The Pillars each have a uniform superlattice orientation, i.e., consisting of a single supercrystalline domain. SPs 1 and 2 are heat-treated, while SP 3 is tested both before and after heat treatment. After the X-ray scattering experiment, the Pillars and SPs (in the cross-linked state, HT) are also tested mechanically via microcompression. Finally, based on the findings that emerged for the heat-treated materials via X-ray analysis, a grain boundary between supercrystalline domains, extracted in the bulk samples also used for the Pillars, is analyzed via in situ STEM.

The enhancement of the mechanical properties resulting from the organic cross-linking is confirmed by the microcompression tests. Data obtained from loading–unloading cycles during compression of Pillars and SPs (see Methods) show a mainly linear elastic deformation behavior, with an average strength of ∼500 MPa for the Pillars and an equivalent fracture strength of 300 MPa for the SPs (calculated as applied load divided by the equatorial cross-section of the sphere). All data are shown in SI section 2. Even though for SCNCs of analogous compositions even higher strength values have been reported,^5,21^ these values are still remarkably high. Their slight decrease compared to previous studies^5,21^ is attributed to the potential presence of damage or misalignments with respect to the applied load direction, due to the multiple microsample transfers and manipulations for the X-ray analysis and the mechanical tests.

### X-ray Scattering Analysis: Superlattice Deformation with Uniaxial Pressing

By means of rotation of the sample by 180° in the X-ray beam, we measured the scattered intensity in 3D reciprocal space for all samples (see SI section 3, Figures S3–S8). An example of the scattering intensity distribution for sample Pillar 1 is shown in Figure 2a, and the corresponding distributions for other Pillars are shown in the SI, Figures S4 and S6. It contains several Bragg peaks, which, by the relative angular positions, can be attributed to an *fcc* structure in real space. The peaks with the lowest *q*-value belong to the 111_*fcc*_ peak family of an *fcc* structure. The corresponding directions of four of them are indicated in Figure 2a. Using their angular positions, one can estimate the unit cell orientation in real space, as shown in Figure 2b. In addition to the Bragg peaks, there is diffuse scattered intensity at lower *q*-values. The measured 3D diffraction patterns contain “flares” of intensity in the horizontal plane of reciprocal space that originate from scattering on the pillar walls. One should also note the splitting of the “flares”, as the opposite pillar walls are not perfectly parallel. Their directions **n**_**1**_, **n**_**2**_, and **n**_**3**_ coincide with the normal vectors to the pillar walls, as shown in Figure 2c. This allows one to detect the orientation of the unit cell with respect to the pillar walls, as shown in Figure 2b.

**Figure 2:**
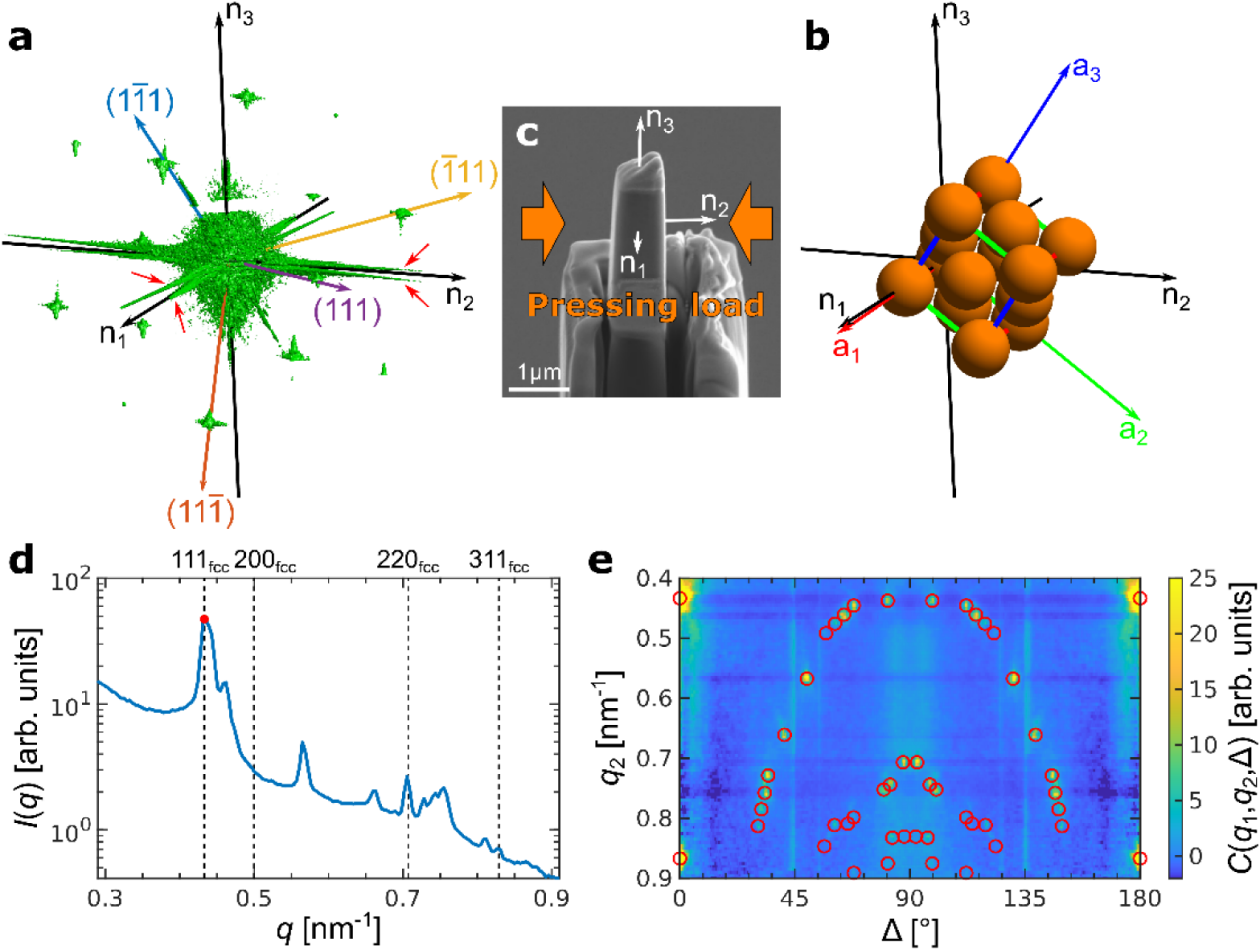
Angular X-ray Cross-Correlation Analysis (AXCCA) of SCNC Pillars. (a) An isosurface of the measured scattered intensity distribution in 3D reciprocal space for Pillar 1. Four (111)_*fcc*_ directions of the reciprocal lattice of a distorted *fcc* lattice are indicated by colored arrows, as well as the normal vectors **n**_**1**_, **n**_**2**_, and **n**_**3**_ to the pillar walls deduced from the intensity “flares” orientation. The red arrows indicate splitting of the intensity “flares” associated with the nonperfectly parallel pillar walls. (b) Orientation of a distorted *fcc* unit cell with respect to the pillar walls in real space. (c) SEM image of the same pillar with indicated directions **n**_**1**_, **n**_**2**_, and **n**_**3**_. The uniaxial stress direction applied during the sample preparation is also indicated. (d) Azimuthally averaged intensity profile of the 3D scattered intensity of Pillar 1, with the red point indicating *q*_1_ = 0.435 nm^–1^, used for the calculation of the cross-correlation functions (CCFs). The peak positions of an ideal *fcc* structure are indicated by vertical dashed lines. (e) CCFs *C*(*q*_1_,*q*_2_,Δ), calculated for *q*_1_ (red point in (d)) and *q*_2_ in the range of 0.4–0.9 nm^–1^, stacked along the vertical axis *q*_2_, with the peak positions for the optimized unit cell parameters marked by red circles.

However, the average azimuthal profile of the scattered intensity distribution, shown in Figure 2d, does not correspond perfectly to an *fcc* superlattice and contains many additional peaks. To extract the unit cell parameters, we applied the AXCCA technique to the collected 3D X-ray scattering data (see Methods and Ref. 60 for details).^64^ It is assumed that the superlattice has the lower-symmetry primitive triclinic structure with parameters *a′*, *b′*, and *c′* and angles between them α′, β′, and γ′. The peak at *q*_1_ ≈ 0.435 nm^–1^ is attributed to the 100 reflection (primitive unit cell). The cross-correlation functions (CCFs) are then calculated for the intensity taken at this *q*_1_ and all other *q*_2_ momentum transfer values in the range 0.4–0.9 nm^–1^. The resulting CCFs *C*(*q*_1_,*q*_2_,Δ) are shown in Figure 2e. There are several peaks at different *q*_2_ values and different angles Δ, highlighted with red circles in the same Figure 2e. Each peak corresponds to a vector **g**_**2**_ of the reciprocal lattice that has a length *q*_2_ = |**g**_**2**_| and relative angle Δ with respect to the vector **g**_**1**_ = **b**_**1**_ with the length *q*_1_ = |**g**_**1**_| ≈ 0.435 nm^–1^. The unit cell parameters are obtained by optimization to fit the experimental peak positions, with the mean value of the experimental CCFs at the calculated peak positions used as a metric, as shown in SI section 3.

The superlattice unit cell parameters of all tested pillars are listed in Table 1. It is immediately noticeable that all values are very close to those of an *fcc* structure (for which *a′* = *b′* = *c′* and α′ = β′ = γ′ = 60°), yet well separated from them, supporting the description of the lattice as a “distorted *fcc*” with the parameters *a*, *b*, *c* and α, β, γ, which are also given in Table 1.

**Table 1:** Unit Cell Parameters of the Pillar Samples Extracted by Angular X-Ray Cross-Correlation Analysis (AXCCA)

	Primitive unit cell parameters						Face-centered unit cell parameters					
Sample	*a*′, nm	*b*′, nm	*c*′, nm	α′, °	β′, °	γ′, °	*a*, nm	*b*, nm	*c*, nm	α, °	β, °	γ, °
**Pillar 1 (HT)**	16.8 ± 0.2	17.0 ± 0.5	17.8 ± 0.3	58.7 ± 1.8	60.8 ± 0.7	68.5 ± 1.1	22.2 ± 0.3	26.5 ± 0.7	25.6 ± 0.6	93.8 ± 2.4	91.1 ± 2.0	91.7 ± 1.8
**Pillar 2 (HT)**	17.1 ± 0.2	17.4 ± 0.3	17.8 ± 0.4	57.0 ± 1.6	63.5 ± 0.8	60.9 ± 0.8	23.6 ± 0.4	26.2 ± 0.4	24.3 ± 0.4	93.3 ± 1.5	88.9 ± 1.6	89.0 ± 1.5
**Pillar 3 (before HT)**	18.2 ± 0.5	16.9 ± 0.5	17.0 ± 0.3	61.3 ± 1.8	58.1 ± 0.6	61.3 ± 1.5	24.5 ± 0.8	23.7 ± 0.9	25.8 ± 0.6	93.1 ± 2.5	86.8 ± 2.3	91.1 ± 2.0
**Pillar 3 (after HT)**	18.0 ± 0.5	16.8 ± 0.4	16.9 ± 0.4	61.5 ± 1.9	58.4 ± 0.8	61.6 ± 1.5	24.4 ± 1.0	23.7 ± 0.8	25.6 ± 0.7	93.1 ± 2.4	87.0 ± 2.7	91.1 ± 2.6

Interestingly, a pattern emerges: all micropillars have one primitive unit cell parameter close to 18 nm, while the other two are closer to 17 nm and an angle smaller than 60°, with the other two being larger instead. This suggests consistency in the superlattice distortion mechanism. Previous studies have shown that the uniaxial pressing step (with confinement in a rigid die) can lead to superlattice anisotropies.^18^ Here, the analysis of the orientation of the superlattice within each micropillar and with respect to the applied pressing load confirms this effect; see Figure 2b,c. This is especially clear in two Pillars (1 and 2), which show the same superlattice orientation, and more specifically the [01̅1]_*fcc*_ axis oriented parallel to the vertical axis of the pillars, and the [100]_*fcc*_ and [011]_*fcc*_ axes in the pillars’ cross-sectional plane. The superlattice has the shortest unit cell constant and is thus compressed most along the [100]_*fcc*_ axis, and secondarily along the [011]_*fcc*_ one. Both of these axes lie in the cross-sectional plane of the pillar, while the dimension along the pillars’ vertical axis is the largest one. One should also note that the largest angle α is opening toward the [011]_*fcc*_ direction. Since the pillars were extracted from an axial cross-section of the bulk cylindrical pellets, the vertical direction of each pillar is oriented perpendicular to the pressing load, thus confirming that the pressing step leads to a superlattice distortion. The superlattice is stretched in the direction perpendicular to the pressing load. Indeed, the SCNCs have been shown to allow for compaction and superlattice stretching due to the presence of free volume, detected in previous studies via both TEM and positron annihilation lifetime spectroscopy (PALS).^56,81^

Pillar 3 has, instead, a superlattice orientation that leads to a less evident effect of the pressing step, although even there the angles α and γ opening in the horizontal plane are bigger than the β opening toward the vertical direction (see SI, Figure S6). This pillar was, additionally, analyzed via X-rays both before and after the cross-linking-inducing heat treatment (HT). It emerges that, even though the organic phase undergoes significant changes and is partially removed during this step,^25,56^ the superlattice does not shrink significantly. As shown in Table 1, the superlattice parameters show only a very slight decrease in their average value, while the average angle values show a small increase, leading to negligible changes given the measurement error.

### X-ray Scattering Analysis: Stacking Faults in Supraparticles

The SPs feature an undistorted *fcc* superlattice since they do not undergo a pressing step. However, the measured X-ray scattering intensity distribution, shown in Figure 3a for the sample SP 1, contains not only the corresponding Bragg peaks, as expected. There are also rod-like features known as Bragg rods,^82^ which indicate here the presence of stacking faults in the structure. Note that while these kinds of rods could also potentially emerge from the tested samples’ planar truncation, such as the Pillars’ walls, they are also detected in the SPs, which have minor levels of truncated areas, and hence, we can conclude that stacking faults are the main cause leading to their appearance. For close-packed crystals, the Bragg rods are continuous intensity modulations along the *h*·**b_1_** + *k*·**b_2_** + *l*·**b_3_** lines, where *h *– *k* ≠ 3*n*; *h*, *k*, *n* ∈ Z and *l* ∈ R , and **b**_**1**_, **b**_**2**_, and **b**_**3**_ are the reciprocal basis vectors of the corresponding *hcp* lattice. The intensity modulation along these peaks is dependent on the stacking order.

**Figure 3:**
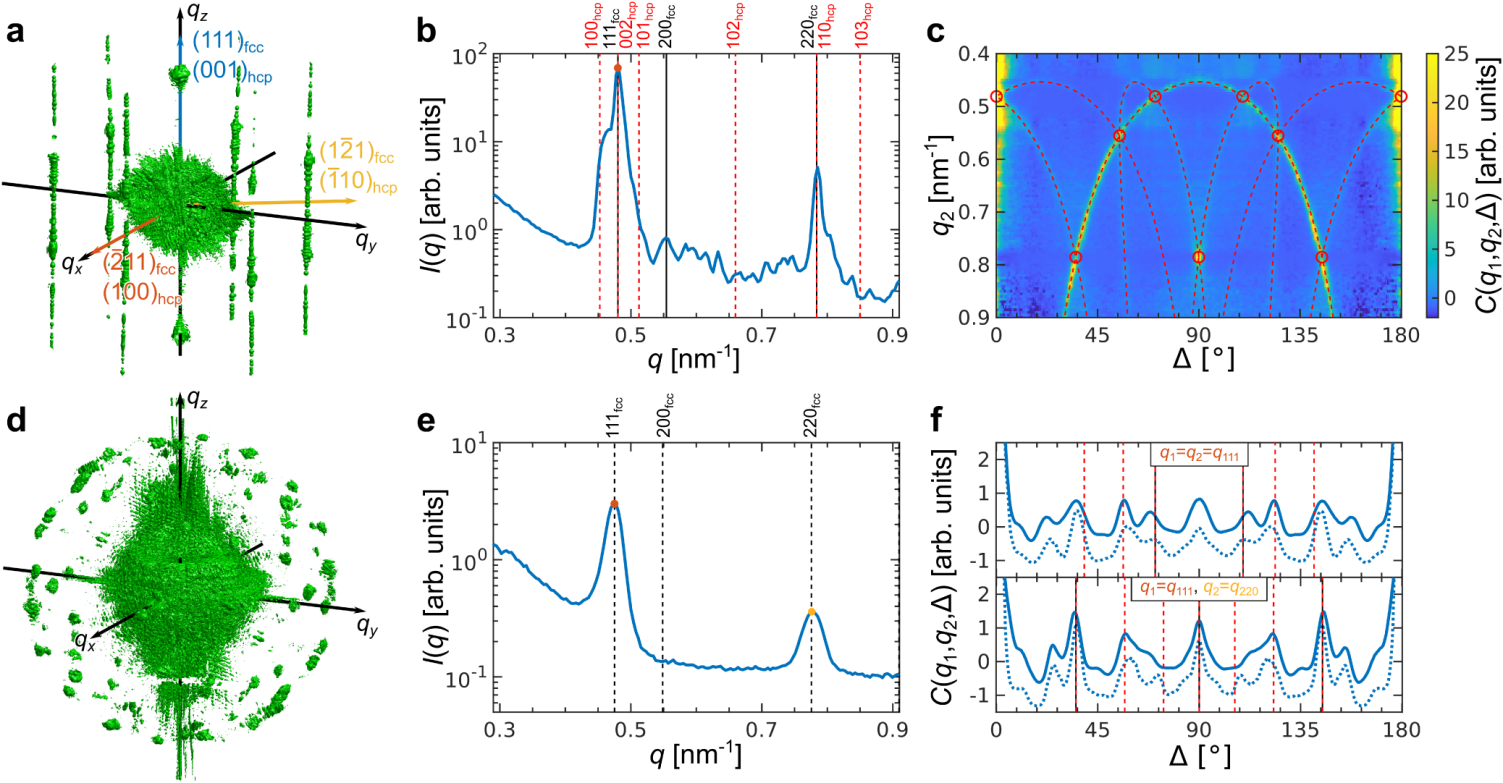
Angular X-ray Cross-Correlation Analysis (AXCCA) of supraparticles (SPs). (a) An isosurface of the measured scattered intensity distribution in 3D reciprocal space for SP 1. The (111)_*fcc*_/(001)_*hcp*_ reciprocal direction corresponding to the stacking direction as well as the (2̅11)_*fcc*_/(100)_*hcp*_ and (12̅1)_*fcc*_/(1̅10)*_hcp_* reciprocal directions constituting the basis in hexagonal close-packed planes are shown with colored arrows. The Bragg rods are an indication of the presence of stacking faults. (b) Averaged radial profile of the 3D scattered intensity of SP 1, with the red point indicating *q*_1_ = 0.480 nm^–1^, used for the calculation of the CCFs. The peak positions corresponding to *fcc* and *hcp* structures are indicated with black and red dashed lines, respectively. The SP structure is thus a random *hcp* (*r-hcp*)^67,68^ containing both *fcc* and *hcp* stacking motifs. (c) CCFs *C*(*q*_1_,*q*_2_,Δ) calculated for *q*_1_ indicated in (a) and *q*_2_ in the range 0.4–0.9 nm^–1^. The CCFs are shown as heat maps stacked along the vertical axis *q*_2_. The peak positions for the optimized nearest-neighbor distance *d*_*NN*_ corresponding to the maximum of correlation are marked with red circles for the *fcc* structure and the red dashed lines for the correlations with the Bragg rods from an *r-hcp* structure. The “arcs” of intensity originate from the correlation between the 111_*fcc*_ reflections and the Bragg rods of the 10*l*_*hcp*_ family. (d) An isosurface of the measured scattered intensity distribution in the 3D reciprocal space for SP 2. (e) Averaged radial profile of the 3D scattered intensity of SP 2, with the red point indicating *q*_111_ = 0.475 nm^–1^ and the yellow point indicating *q*_220_ = 0.775 nm^–1^, used for the calculation of the CCFs. The peak positions for an *fcc* structure are indicated with black dashed lines. (f) CCFs *C*(*q*_1_,*q*_2_,Δ) calculated for *q*_1_ = *q*_2_ = *q*_111_ = 0.475 nm^–1^ (top) and *q*_1_ = *q*_111_ = 0.475 nm^–1^, *q*_2_ = *q*_220_ = 0.775 nm^–1^ (bottom). The solid lines are calculated for the experimental intensity distribution, and the dashed ones for the simulated. Several peaks here cannot be attributed to a single *fcc* lattice, and indeed SP 2 is found to have an anti-Mackay structure (see Figure 1e, SI Section 4, and Figure S11).

The corresponding average radial profile shown in Figure 3b contains two bright peaks that can be attributed to the 111_*fcc*_ and 220_*fcc*_ reflections of the *fcc* structure. An additional intensity on the left side of the 111_*fcc*_ reflection can be attributed to the 100_*hcp*_ reflection of an *hcp* structure with the same nearest-neighbor distance as expected for a close-packed structure with stacking faults. Small peaks between the 111_*fcc*_ and 220_*fcc*_ reflections originate from the intensity distribution along the Bragg rods. Thus, the structure of the SPs can be characterized as a random hexagonal close-packed (*r-hcp*)^67,68^ structure containing both *fcc* and *hcp* stacking motifs. For SP 3, we observe a similar 3D intensity distribution and azimuthal profile, as shown in Figure 4a,b.

**Figure 4:**
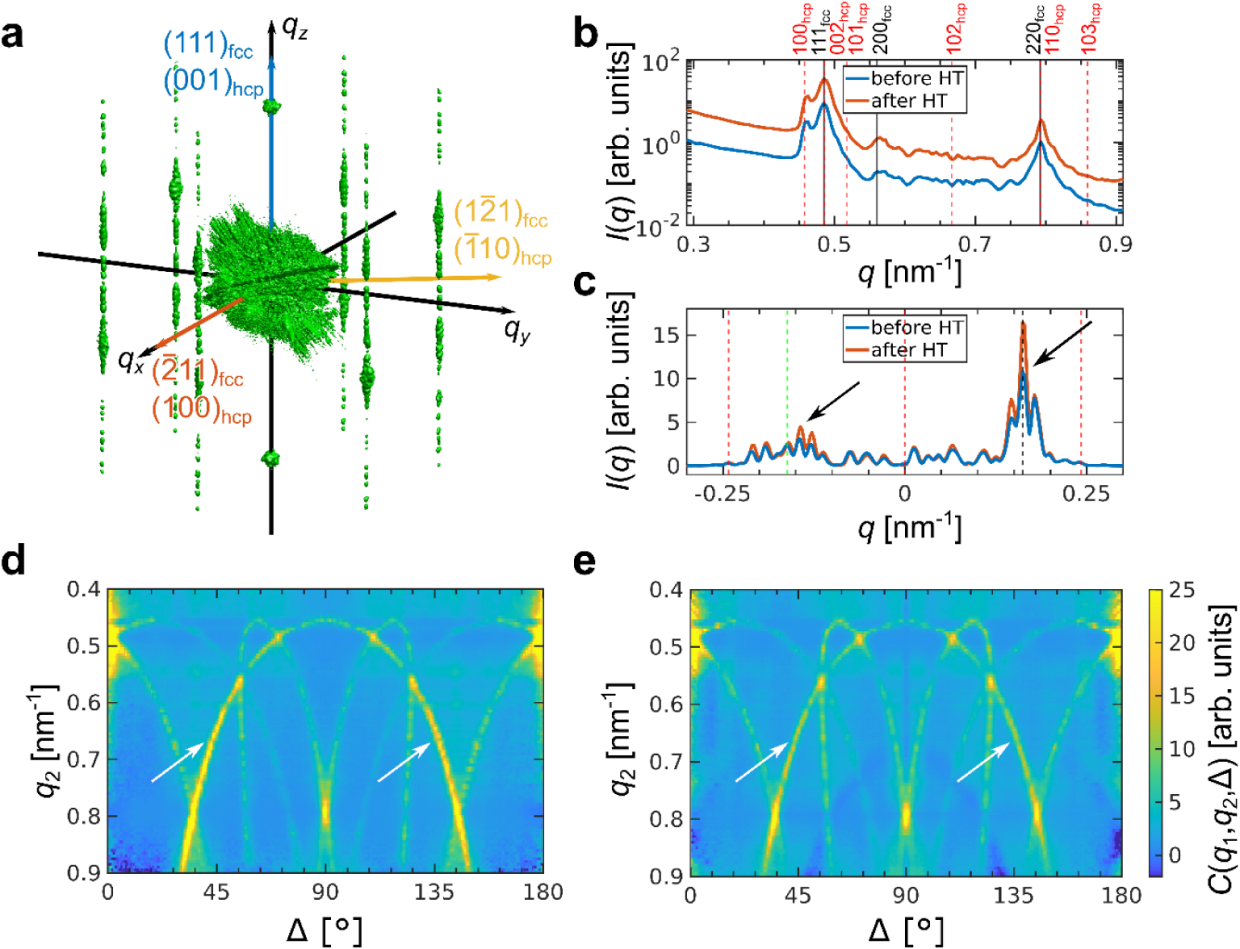
Angular X-ray Cross-Correlation Analysis (AXCCA) of an SP before and after heat treatment. (a) An isosurface of the measured scattered intensity distribution in 3D reciprocal space for SP 3. The (111)_*fcc*_/(001)_*hcp*_ reciprocal direction corresponding to the stacking direction as well as the (2̅11)_*fcc*_/(100)_*hcp*_ and (12̅1)_*fcc*_/(1̅10)_*hcp*_ reciprocal directions constituting the basis in hexagonal close-packed planes are shown with colored arrows. The Bragg rods are an indication of the presence of stacking faults. (b) Averaged radial profiles of the 3D scattered intensities of SP 3 before (blue line) and after (red line) heat treatment. The profiles are shifted vertically for the sake of clarity. The peak positions corresponding to *fcc* and *hcp* structures are indicated with black and red dashed lines, respectively. (c) Averaged intensity profile along the 10*l*_*hcp*_ Bragg rods before (blue line) and after (red line) heat treatment. The peak positions corresponding to the *fcc* and *hcp* structures are indicated with black and red dashed lines, respectively, and the twinned *fcc* structure is indicated with green dashed lines. (d,e) CCFs *C*(*q*_1_,*q*_2_,Δ) calculated for *q*_1_ corresponding to the 111_*fcc*_ Bragg peak and *q*_2_ in the range of 0.4–0.9 nm^–1^ before (d) and after (e) heat treatment. The CCFs are shown as a heat map stacked along the vertical axis *q*_2_. (c–e) point at the thermal annealing of the stacking faults via the intensity profiles along the 10*l*_*hcp*_ Bragg rods shown in (c) and the lower intensity of the central yellow “arc” in (e) (after heat treatment) compared to (d), as marked by the arrows. Note that the dark blue arc in (e) is an artifact originating from the fact that we do not measure the whole reciprocal space.

To refine the identification of the SP structure, we applied AXCCA to the 3D intensity distribution. The CCFs were calculated for the intensities taken at *q*_1_ corresponding to the 111_*fcc*_ reflection and all other momentum transfer values at *q*_2_ in the range of 0.4–0.9 nm^–1^. The resulting CCFs are shown in Figures 3c and 4d. Differently from the Pillars, the maps for the SPs contain not only peaks but also “arcs” of intensity (in yellow and marked with red dashed lines in the figures). They originate from the correlation between the 111_*fcc*_ reflections and the Bragg rods of the 10*l*_*hcp*_ family.

The SP unit cell parameters were optimized with the same procedure as for the triclinic structure of the Pillars, even though, in this case, in the absence of superlattice distortion, there is only one parameter to be determined: the nearest neighbor distance *d*_*NN*_ between adjacent NPs. The optimized *d*_*NN*_ values are summarized in Table 2. One can calculate the unit cell parameters $a_{fcc}=\sqrt{2}d_{NN}$ for the *fcc* and $a_{hcp}=d_{NN},c_{hcp}=\sqrt{8/3}d_{NN}$ for the *hcp* structures from the *d*_*NN*_ values.^30^ The nearest neighbor distance is consistently ∼16 nm, again showing no detectable superlattice shrinkage associated with the heat treatment. For the *hcp* structure, both 100_*hcp*_ and 002_*hcp*_ peaks are detected, and no deviations from the ideal ratio are observed. Based on these features, the structure of the SPs can be described as *r-hcp* with prevalent *fcc* stacking motifs. No peaks that can be attributed solely to *hcp* domains are observed, but due to the stacking faults, there can be *hcp* areas with the thickness of a few NP layers.

**Table 2:** Nearest-Neighbor Distances and Unit Cell Parameters of SPs as Extracted by AXCCA

Sample	Nearest-neighbor distance *d*_*NN*_, nm	*fcc* unit cell parameter *a*_*fcc*_, nm	*hcp* unit cell parameter *a*_*hcp*_, nm	*hcp* unit cell parameter *c*_*hcp*_, nm
**SP 1 (HT)**	16.0 ± 0.1	22.6 ± 0.2	16.0 ± 0.1	26.1 ± 0.2
**SP 2 (HT)**	16.2 ± 0.2	22.9 ± 0.3	-	-
**SP 3 (before HT)**	15.9 ± 0.2	22.5 ± 0.3	15.9 ± 0.2	26.0 ± 0.3
**SP 3 (after HT)**	15.9 ± 0.2	22.5 ± 0.3	15.9 ± 0.2	26.0 ± 0.3

SP 2 has a very distinctive structure. The 3D intensity distribution shown in Figure 3d contains many Bragg peaks at the same *q*-value, which cannot be attributed to a single crystalline structure. On the other hand, the radial profile shown in Figure 3e contains two peaks at *q* = 0.475 nm^–1^ and 0.775 nm^–1^ that can be attributed to 111_*fcc*_ and 220_*fcc*_ reflections of an *fcc* structure with *a*_*fcc*_ = 22.9 ± 0.5 nm (*d*_*NN*_ = 16.2 ± 0.3 nm). The CCFs calculated for these two *q*-values are shown in Figure 3f. They contain several peaks that cannot be attributed to a single *fcc* lattice. Given its size, this supraparticle is indeed expected to have the so-called anti-Mackay structure,^78^ which consists of many mutually twinned *fcc* domains, also in line with SEM observations (see Figure 1e). The geometric calculation of the expected correlation peak positions for the anti-Mackay structure is quite cumbersome due to the complex relative orientations of the multiple twinned domains. Instead, we simulated the 3D scattered intensity distribution for an SP with the anti-Mackay structure and similar size as described in the SI, Section 4. Comparison of the CCFs calculated for the simulated intensity distribution with the experimental ones shown in Figure 3f confirms the anti-Mackay structure of SP 2. We refer to Supporting Information, Figure S11 for the full 2D experimental and simulated CCF maps. It is worth mentioning here that SPs can feature a variety of superstructures depending on their size with respect to the size of the constituent NPs, as highlighted in several studies.^38,69,78,83–85^

### Thermal Annealing of Planar Defects

The heat treatment has been shown to induce no detectable shrinkage for both Pillars and SPs (Tables 1 and 2). However, it plays an important role in the mobility, rearrangement, and migration of superlattice defects. We show in the following that annealing the SPs leads to the healing of the stacking faults, while in the bulk samples, heating leads to rearrangement of a supercrystalline grain boundary. The scattered intensity distribution of SP 3 before heating, shown in Figure 4a, is similar to that of SP 1 (Figure 3a). It contains Bragg peaks and Bragg rods and can also be characterized as an *r*-*hcp* structure with *fcc* and *hcp* stacking motifs and many stacking faults. The radial profile shown in Figure 4b contains the corresponding Bragg peaks.

Remarkably, the heat treatment does not lead to major changes in the 3D intensity distribution or the azimuthal profile. However, the heat treatment does lead to the redistribution of intensities in the CCF maps of the same SP 3 before and after heat treatment (Figure 4d,e). One can see that the central “arc” has higher intensity before heat treatment than afterward. This “arc” comes from the correlations of the 10*l*_*hcp*_ Bragg rods with the 111_*fcc*_/002_*hcp*_ stacking-independent Bragg peaks that are normal to the hexagonal planes. Other “arcs” originate from the correlations between the Bragg rods and the 111_*fcc*_ peaks that are not normal to the hexagonal planes and thus originate purely from *fcc* motifs. The intensity of the stacking-independent peaks is supposed to be constant, but the CCFs are normalized by the total intensity. It thus emerges that the intensities of the stacking-dependent 111_*fcc*_ peaks have risen upon heat treatment, indicating an increased ratio of the *fcc*/*hcp* domains. This effect is also confirmed by higher intensities at the peak positions that are characteristic for an *fcc* structure in the CCF maps, and it can be seen in the intensity profiles along the 10*l*_*hcp*_ Bragg rods shown in Figure 4c. Thus, we conclude that the stacking faults are healed by means of heat treatment.

To verify this effect on the stacking faults, the temperature-dependent evolution of a SCNC with *hcp* and *fcc* domains was studied by using all-atom molecular dynamics (MD) simulations. The simulated SCNC consists of magnetite NPs, which are 4 nm in diameter and functionalized with oleic acid molecules. These have been used to build and equilibrate an *fcc* SCNC. From the equilibrated *fcc* SCNC, an “ABCABCABABAB” system was generated. This represents the shortest sequence that hosts both *fcc* and *hcp*, while allowing, in principle, for a transition to a pure *fcc* or *hcp* crystal structure. The generated SCNC contains 24 functionalized NPs with approximately 400000 atoms; see Figure 5a. The system size and simulation time were selected to be the minimum required to observe the healing of the stacking faults in the supercrystalline lattice.

**Figure 5:**
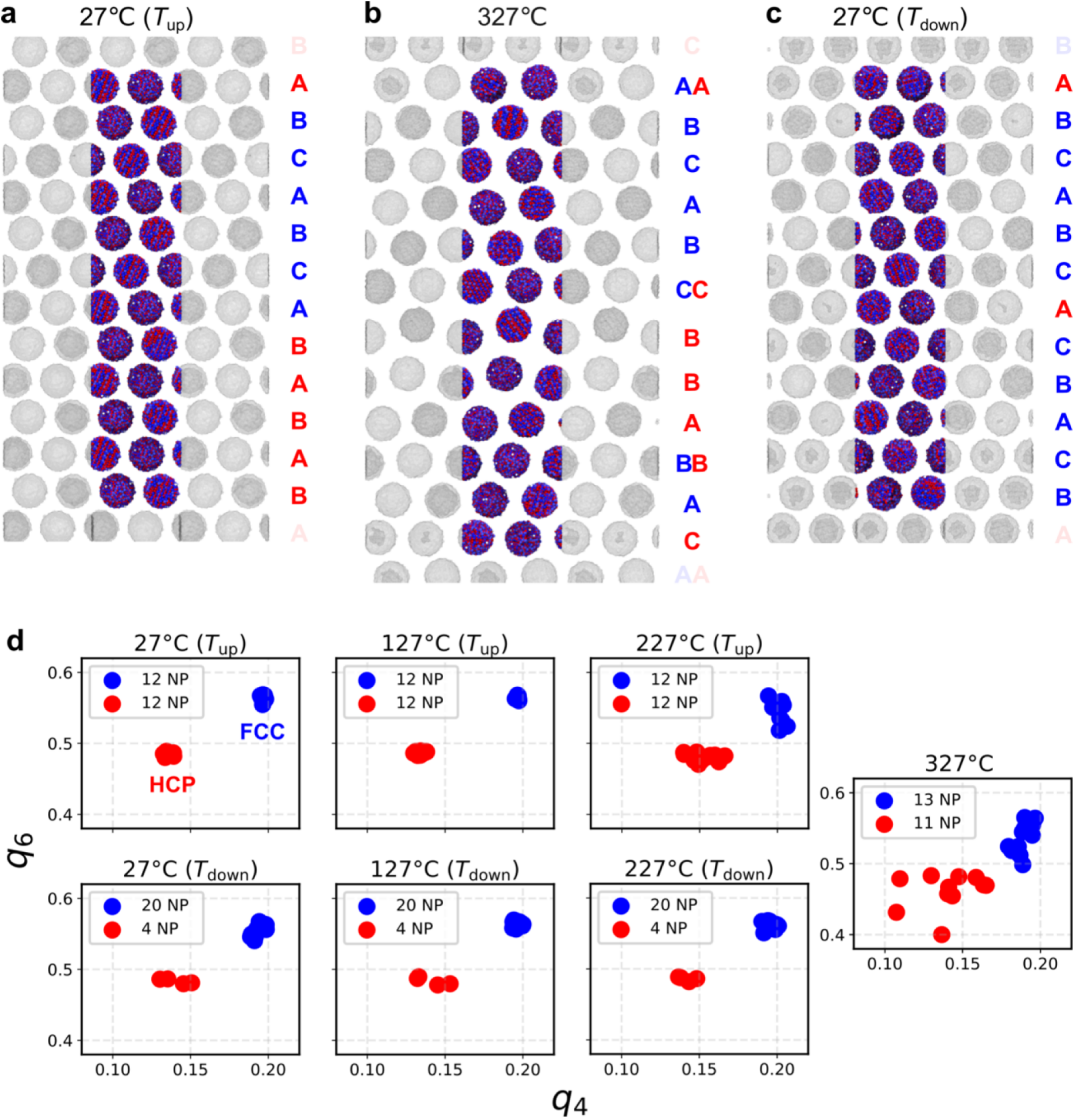
All-atom simulation of the temperature-dependent evolution of a *hcp*/*fcc* supercrystalline nanocomposite (SCNC). The temperature and whether the system is heated (*T*_up_) or cooled (*T*_down_) are specified in each plot title: (a) at 27 °C (*T*_up_); (b) at 327 °C; and (c) at 27 °C (*T*_down_). The stacking sequence with the corresponding structure’s color code is shown on the right. For clarity of representation, the oleic acid molecules are not shown. The simulation boxes are highlighted, and each layer contains two NPs. (d) Steinhardt bond order parameters, *q*_4_ and *q*_6_, of the system containing 24 oleic acid-functionalized magnetite NPs. *fcc*-like and *hcp*-like NPs are indicated by blue and red dots, respectively. The amounts of the corresponding NPs are given in the legend.

The system was heated for 75 ns from 27 to 327 °C, denoted by *T*_up_, at a constant pressure of 1 atm in an *NpT* ensemble. Next, the system was held at 327 °C for 10 ns and then cooled to 27 °C, denoted by *T*_down_, over 75 ns. Steinhardt bond order parameters,^86^ particularly the *q*_4_ and *q*_6_ parameters, were employed to determine the local supercrystalline structure of the NPs, namely for distinguishing between *fcc* and *hcp* local ordering. The *q*_4_ and *q*_6_ parameters are the necessary and sufficient ones required to distinguish *fcc* and *hcp* structures. At 27 °C (*T*_up_), *q*_4_ and *q*_6_ bond order parameters indicate that 12 NPs are in an *fcc* and 12 in an *hcp* configuration (see Figure 5d). Both *fcc* and *hcp* NPs remain relatively stable close to 327 °C. At around 327 °C, the distances between NPs have increased, and the *hcp* NPs start to reorganize, while the *fcc* NPs remain in their initial configuration. This reorganization happens through migration of several single NPs, which, thanks to the energy provided via heating and the resulting increased distance among them, start wiggling and ultimately rotate into their new positions. After cooling to 27 °C (*T*_down_), the reorganization of *hcp* NPs becomes more apparent, with 8 out of 12 transitioning to an *fcc* structure. A closer look at the system at 27 °C (*T*_down_) (see Figure 5c) reveals that the *hcp* signal in the *q*_4_-*q*_6_ analysis stems from twin boundaries in the *fcc* crystal structure, resulting in the stacking order of “(A)­BCABC­(A)­CBACB”, where stacking faults are highlighted with brackets.

These results show that at the applied temperature, the SCNC can reorganize from an *hcp* arrangement into *fcc*, in very good agreement with the experimental observations. It should be noted that due to the relatively small size of the simulated system, containing only 24 NPs, finite size effects in the simulations cannot be ruled out completely, which might affect the presence and particularly the density of defects such as twin boundaries. Nevertheless, the very good agreement with the experimental AXCCA results is a confirmation of the appropriateness of the MD simulation framework for the purposes of this study. The combination of AXCCA and MD analysis is then valuable in providing complementary information: AXCCA allows monitoring the evolution of the superlattice in the entire SP, while MD provides insights into how the NPs achieve the migration leading to stacking fault healing. Additional insights can potentially come in future work via electron tomography, which can provide stacking sequence and *hcp/fcc* ratios before and after annealing, even though in smaller supercrystalline domains compared to those analyzed via AXCCA.

In general, the occurrence of stacking faults and twin boundaries in SCNC can be expected and was observed in iron oxide–oleic acid bulk SCNC.^5^ Remarkably, however, their rearrangement and healing—to the best of the authors’ knowledge—had not yet been detected. Annealing of twins and their migration is a well-known phenomenon, even though not fully understood, in polycrystalline metallic systems at temperatures above the recrystallization ones,^87^ and the healing of stacking faults has also been observed in several crystalline materials.^88,89^ Here, however, we observe healing of these planar defects present in the supercrystals as an outcome of the SP formation, at significantly larger length scales. The transition from *hcp* to *fcc* motifs has been predicted in colloidal crystals of hard spheres, in months-to-years timeframes.^90^ What stands out here is the combined experimental and numerical observation of this transition happening in supercrystals containing organic ligands, within hours, and during a heat treatment that also leads to the cross-linking of the organic ligands, which induces a multifold strengthening of the SCNCs.

In the bulk samples from which the Pillars have been extracted, 2D defects are also expected in the form of stacking faults and intersupercrystalline “grain” boundaries.^56^ The temperature-induced migration of these kinds of 2D defects is then demonstrated with an in situ STEM heating experiment on a supercrystalline grain boundary from a bulk SCNC, shown in Figure 6. A thin lamella was obtained via FIB milling from an area that included such a boundary in a non-HT bulk SCNC. The boundary was oriented close to edge-on condition,^74^ such that the defects along it could be discerned (with minor distortions given the deviation from edge-on condition). Figure 6 presents high-angle annular dark field (HAADF) STEM micrographs, demonstrating disconnections along the boundary that is oriented close to edge-on condition (see also SI section 6 and Figure S14). Disconnections are interfacial line defects with a step and/or a dislocation component,^91–96^ and they have so far been detected in polycrystalline materials, such as at general and high-symmetry grain boundaries in SrTiO_3_.^66^ and at grain and phase boundaries in β-Yb_2_Si_2_O_7_.^76,77^ Anisotropic motion of disconnections has also been detected during in situ STEM heating experiments, indicating that such motion is the mechanism of grain boundary migration in both general and high-symmetry grain boundaries.^94^

**Figure 6:**
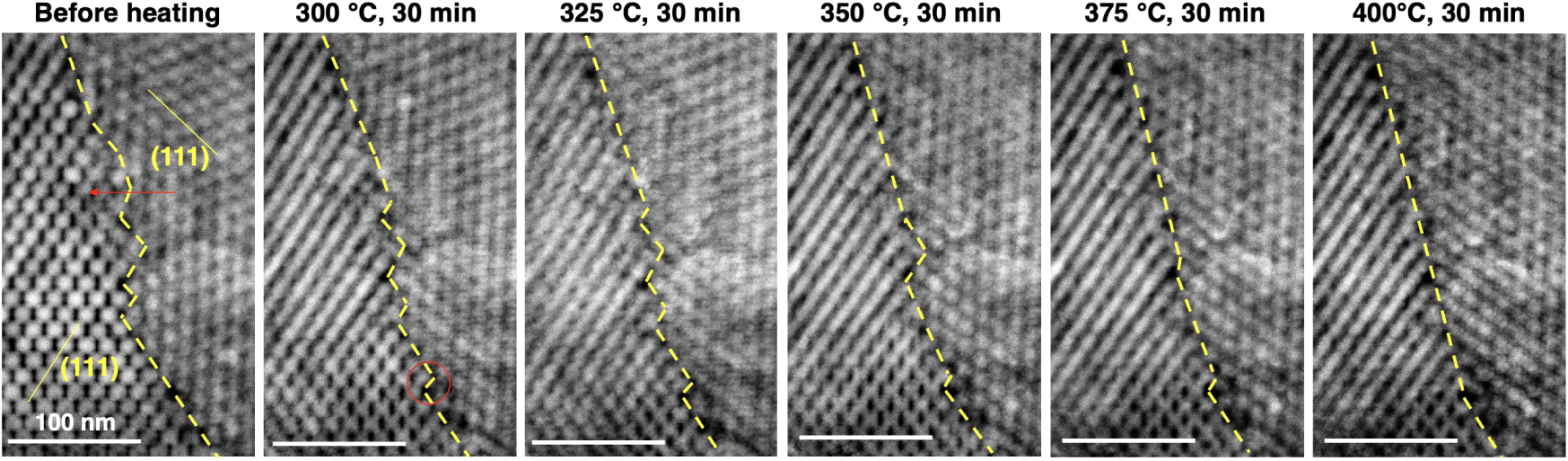
HAADF STEM micrographs of an in situ heating experiment of a supercrystalline grain boundary. Disconnections with grain boundary and step planes parallel to {111}_*fcc*_ planes are demonstrated along a supercrystalline grain boundary from bulk SCNCs. Anisotropic motion of disconnection along with grain boundary migration is noted upon heating. The area is observed edge-on with zone axis [01̅1]_*fcc*_ (see also Figure S14). The dashed yellow lines mark the grain boundaries and step planes.

Here, the grain boundary and step planes are parallel to specific crystallographic planes, i.e., {111}_*fcc*_ superlattice planes, throughout the heating experiment, indicating their anisotropy in the case of this boundary. Even though we consider here a single general supercrystalline grain boundary, the anisotropy of the ledges and terraces at the fracture surfaces of SCNCs (Figure 1), as well as the uniform anisotropy of disconnections and ledges and terraces at interfaces in various conventional polycrystalline systems, suggests that other supercrystalline grain boundaries would exhibit the same anisotropy.

Most importantly, upon heating, the disconnections migrate along the boundary, resulting in a decrease in the local number of steps and the boundary becoming progressively straighter. Whether the change in the number of steps is local or reduced throughout the entire boundary, the step height appears to remain similar, such that step bunching is not noted. These results extend the concept of anisotropic disconnection motion being the mechanism of grain boundary migration, and they provide experimental evidence beyond previously studied polycrystalline systems,^74,75,92–94^ i.e., in SCNCs with lattice parameters 2 orders of magnitude larger than those of conventional crystals, and over longer time scales (minutes instead of seconds). Given the anisotropy of the ledges and terraces in SCNCs, as well as the anisotropy of disconnections and ledges and terraces at interfaces in polycrystalline systems, it is expected that other general supercrystalline grain boundaries would indicate similar anisotropic behavior of disconnections.

Note that a very significant degradation of the organic phase is typically observed at temperatures above 350 °C,^17,21^ and thus NP sintering is expected in the last in situ heating stages (at 375 and 400 °C). On the other hand, the TEM lamella is fixed at its edges for the in situ study, hampering the shrinkage. Figure S14 shows the estimated distance between close-packed {111}_*fcc*_ superlattice planes with increasing temperature, both from the local TEM measurement and from global SAXS data on the whole bulk SCNC from which the lamella is extracted. For the TEM case, since both abutting grains are not fully aligned in the zone axis but rather near it, and minor sample tilts as well as sample bending occur during the experiment, the interplanar distances cannot be determined with high precision but only estimated. The threshold value below which NPs start to sinter (12.2 nm) is obtained with the assumption that the oleic acid on the NP surfaces is completely removed, and the NPs thus come into contact with each other. In all cases, one can see that the interplanar distance remains above the threshold value, corresponding to the onset of necking and sintering. This might suggest that, in addition to the lamella constraint, the high symmetry of the NP *fcc* arrangement and the quasi-constant NP size prevent sintering, even after the pinning effect of the organic ligands is removed.^97^

Both the ex situ AXCCA analysis on SPs and the in situ STEM heating study on a grain boundary in bulk SCNCs then lead to a very interesting finding: the NPs in the SCNCs are able to migrate and heal or reconfigure superlattice defect structures. This phenomenon is thermally activated. One can consider additional potential factors that can play a role in this reconfiguration: the NP size can alter the van der Waals interactions among SCNC building blocks, and the organic functionalization and chemical reaction leading to ligand cross-linking can alter the inter-NP distances, and therefore the NP ability to migrate within the superlattice.^25,29^ The effect of these parameters can be the object of future work, also involving, e.g., systems that are electrostatically stabilized instead of ligand-stabilized. However, the NP migration detected here relies on a temperature increase, as also confirmed by the MD simulations, where temperature changes are the sole driving force leading to the superlattice reconfigurations, and these match very well with the experimental observations. Importantly, the heat treatment is also responsible for the cross-linking of the organic ligands, which in turn is expected to fix the NPs into their superlattice sites, preventing migration. Therefore, the kinetics of the NP migration and cross-linking are expected to be different. Indeed, the MD simulations show that the heat treatment leads to NP migration and stacking fault healing within a time scale of nanoseconds, while the full cross-linking reaction takes up to hours.^25^ The complex radical oxidative polymerization reaction that leads to the ligand cross-linking,^25^ additionally, leads to partial decomposition and rearrangement of the ligands, also offering chances for NP mobility in longer time scales, as observed in the in situ STEM study. An in-depth analysis of the kinetics of these phenomena could be the object of future work.

## Conclusions

This study provides insights into how distinct processing stages—self-assembly, pressing, and heat treatment—affect the formation, evolution, migration, and healing of supercrystalline defects in high-strength inorganic–organic supercrystalline nanocomposites (SCNCs). The combination of AXCCA, MD, and in situ STEM reveals how superlattice distortion, stacking faults, and supercrystalline grain boundaries all contribute to the structural complexity of SCNCs. In bulk pellets, the pressing step leads to a distortion of the otherwise *fcc* superlattice, which becomes stretched in the plane perpendicular to the applied load. Supraparticles obtained via emulsion-templated self-assembly, instead, show *fcc* NP arrangements with the presence of stacking faults and anti-Mackay structures, depending on their size.

Remarkably, we show that annealing at cross-linking-relevant temperatures (∼350 °C) not only strengthens the material but also leads to the migration and healing of planar defects. The stacking faults are found to be partially removed, as also confirmed via MD simulations, while an intersupercrystalline grain boundary is found to migrate via anisotropic disconnection motion. The heat treatment, typically applied to strengthen the SCNCs, is then found to serve the additional role of inducing defect migration and healing. The kinetics of the two mechanisms are thus expected to be different, with defect migration occurring in significantly shorter time scales than ligand cross-linking, as also indicated by the MD simulations, and the complex ligand cross-linking reaction^25^ also offering chances for the NPs to rearrange themselves before becoming fixed into specific superlattice sites.

The demonstrated thermally activated migration of planar defects stands out in the analysis of supercrystals because it does not occur during self-assembly or in low-viscosity films but in a material state that cannot be considered as soft matter. Even before cross-linking, these SCNCs reach bending and compressive strengths beyond 100 MPa. The parallelism with defect migration in crystalline materials thus becomes significantly more apparent and is supported by both numerical and experimental evidence. We envision a variety of new insights on defect migration in crystals based on supercrystalline platforms and the establishment of new concepts for defect engineering in NP assemblies.

## Methods

### Sample Processing

The SCNCs’ building blocks are iron oxide NPs that are surface-functionalized with oleic acid (Fraunhofer CAN GmbH, Hamburg, Germany). The size of the NPs has been assessed via small-angle X-ray scattering (SAXS) of the initial NP suspension in toluene, according to a method reported elsewhere.^25,98^ The NP radius is determined as 7.4 ± 0.8 nm. This is considered to be the radius of the inorganic core, without the organic functionalization, since the latter is markedly less detectable via X-rays, especially when the NPs are still in suspension.

The bulk SCNCs are prepared via self-assembly by the solvent destabilization method.^21^ The NP suspension, with a concentration of 40 g·L^–1^, is filled into an assembly of die and punch with a 14 mm diameter cavity so that the pressing step can immediately follow the self-assembly. It is then placed in a desiccator, where the atmosphere is enriched with ethanol, which serves as a destabilization agent when it slowly diffuses into the suspension. The process lasts approximately 15 days. The self-assembled samples, which at this point are sedimented at the bottom of the die-punch assembly, are recovered by removing the supernatant with a pipet. The SCNCs so obtained are dried for 24 h at ambient conditions and 2 h in vacuum. The pressing step finally follows, by applying 50 MPa via a second punch (in the rigid die) at a temperature of 150 °C. The bulk cylindrical SCNC pellets are 14 mm in diameter and ∼4 mm thick.

The supraparticles were prepared via emulsion-templated self-assembly. A solution of 113 g of polyoxyethylene (20) sorbitan monolaurate (Tween 20, AppliChem, ≥100%), 3.48 g of sorbitan monolaurate (Span 20, Merck, synthesis grade), 6.90 g of sodium dodecyl sulfate (SDS, Chemsolute, ≥98.0%), and 10.6 g of hydroxyethyl cellulose (HEC, Sigma-Aldrich) in 1.50 L of water was prepared in a 2 L round flask via mild stirring at 50 °C until the HEC was dissolved. After allowing it to cool down to room temperature, a suspension of 160 g·L^–1^, oleic acid-stabilized Fe_3_O_4_ nanoparticles (11.5 wt % organic content) in 50 mL cyclohexane (Chemsolute, ≥99.8%) was slowly added to the aqueous solution with a syringe while strongly agitating at 13500 rpm using an Ultra-Turrax (IKA, Germany) for 1 min. The flask was kept open to allow evaporation of the solvent, and the stirring was continued at 60 rpm using a 68 × 24 × 3 mm^3^ PTFE stirrer shaft attached to a mechanical stirrer. The clusters were separated, after 20 h, from the remaining free NPs in the surfactant solution by magnetically decanting the supernatant and redispersing in fresh water. Surfactants were removed by washing the supraparticles 6 times with warm 15% ethanol followed by 6 times with warm 96% ethanol, until the particles agglomerate in polar solvents like water. The cleaned supraparticles were redispersed in ethyl acetate and dispersed on a silicon wafer via spin coating.

The heat treatment to induce the cross-linking of the organic ligands was conducted in a tube furnace, with a hold temperature of 325 °C, a hold time of 18 min, and a heating ramp of 1 °C·min^–1^, under nitrogen (N_2_) atmosphere.

### SEM and FIB

Micrographs of the bulk SCNCs fracture surfaces were obtained via scanning electron microscopy (SEM, Zeiss SUPRA 55-VP, Zeiss, Germany), using a 1–5 kV acceleration voltage, working distance of 4–7 mm, and through-the-lens detector (TLD) or Everhart–Thornley detector (ETD). The micropillars from the bulk samples were fabricated via focused ion beam (FIB) milling with a gallium ion source (FEI Helios NanoLab G3, Thermo Fisher Scientific, Oregon, USA). For the milling process, currents from 21 nA to 1.1 pA for rough cuts and subsequent polishing were used. These parameters are selected based on previous studies on analogous material systems,^15^ which allow us to consider the FIB-induced damage and potential degradation of the organic ligands (in their ultraconfined and often cross-linked state) to be negligible for the purposes of this study. Three of each sample type, micropillars from bulk samples and supraparticles, were transferred onto small pins, providing sufficient elevation to avoid shadowing during the X-ray analysis. The pins were previously tested to ensure sufficient stability at the heat treatment temperature (325 °C), since one supraparticle and one pillar were tested via X-rays both before and after heat treatment. No significant alteration of the pins was detected due to heat treatment. The sample transfer onto the pins and attachment was done in the FIB instrument using a micromanipulator (see, e.g., Figure S1) and ion beam-induced deposition (IBID) of a Pt precursor material via a gas injection system (GIS), respectively.^99^ Micrographs of the prepared micropillars and supraparticles were obtained using a scanning electron microscope under high-vacuum mode, with a 5 kV acceleration voltage, 50 pA beam current, and a working distance of 7 mm and with an Everhart–Thornley detector (ETD).

### X-ray Scattering Experiment

The X-ray scattering experiment was performed at the Coherence Applications beamline P10 at the PETRA III synchrotron source (DESY, Hamburg, Germany). Monochromatic X-rays of 10 keV were focused down to ∼2.5 × 1.9 μm^2^ (horizontal × vertical) at the sample position, completely covering the SCNC samples. The sample pin was fixed on a rotation stage rotating around the vertical axis. At each angular position, the 2D far-field diffraction patterns were recorded by an EIGER X 4M detector positioned 5.0 m downstream of the sample. The sample was rotated by the increment of 1/3° over the range of 180°, and by that, the full 3D diffraction pattern was measured. At each angular position, 5 frames with 1 s exposure each were collected and then averaged. The sample was cooled using a liquid nitrogen jet to avoid radiation damage of the organic ligands stabilizing the NPs, which could induce the NPs’ coalescence and destroy the superlattice ordering.

### AXCCA

Details on the application of the AXCCA technique to a 3D scattered intensity distribution can be found in refs. 59 and 64. The AXCCA is based on the calculation of the cross-correlation function (CCF), defined as

$$C\left(q_{1},q_{2},\Delta \right)=\left\langle Ĩ\left(q_{1}\right)Ĩ\left(q_{2}\right)\delta \left(\frac{q_{1}\cdot q_{2}}{\left|q_{1}\right|\left|q_{2}\right|}-\cos\Delta \right)\right\rangle$$ 1

where $Ĩ\left(q_{1}\right)$ and $Ĩ\left(q_{2}\right)$ are the normalized scattered intensities taken at the momentum transfer vectors **q**_**1**_ and **q**_**2**_, respectively, with Δ being the relative angle between them. Equation 1 is averaged over all angular positions of vectors **q**_**1**_ and **q**_**2**_ with the momentum transfer values *q*_1_ and *q*_2_, respectively. The intensity Ĩ(q) is normalized by the average intensity at the corresponding *q*-value:

$$Ĩ\left(q\right)=\frac{I\left(q\right)-{\left\langle I(q)\right\rangle }_{|q|=q}}{{\left\langle I(q)\right\rangle }_{|q|=q}}$$ 2

When analyzing (super)­crystalline materials, CCFs contain peaks at the relative angles between the Bragg peaks, i.e., the angles between a certain pair of the families of (super)­crystallographic planes corresponding to the reflections at *q*_1_ and *q*_2_. These angles provide additional information on the (super)­crystalline structure compared to the conventional analysis of the azimuthal intensity profiles.

Given a model of the unit cell with the lattice basis vectors **a**_**1**_, **a**_2_, and **a**_**3**_, one can calculate the reciprocal basis vectors **b**_**1**_, **b**_**2**_, and **b**_**3**_, and thus any reciprocal lattice vector **g** *= h·* **b_1_ **+ *k·* **b_2_ **+ *l·* **b**_**3**_. Then, the angles between any (super)­crystallographic planes can be calculated using the dot product of the corresponding reciprocal vectors $\cos\left[\Delta_{ij}\right]=\frac{g_{i}\cdot g_{j}}{\left|g_{i}\right|\left|g_{j}\right|}$ . The angle gives the expected peak position in the CCF *C*(*q*_1_,*q*_2_,Δ) calculated for the scattered intensities at momentum transfer values *q*_1_ = |**g**_*i*_| and *q*_2_ = |**g**_*j*_|. By taking into account the (super)­lattice symmetry, one can calculate all expected positions of the correlation peaks and optimize the unit cell parameters to fit the experimentally obtained peaks. For the SCNC samples considered in this study, CCFs were calculated between the intensities taken at *q*_1_, corresponding to the first bright peak (the exact momentum transfer values *q*_1_ can differ for different samples), and at *q*_2_, varying in the range from 0.4 to 0.9 nm^–1^, with a step size of 0.005 nm^–1^. The resulting CCF maps are then shown in (*q*_2_,Δ)-coordinates.

### In Situ Heating STEM

The procedure of temperature ramping and holding for in situ STEM heating was performed in an FEI Talos F200x (Thermo Fisher Scientific, USA) using an in situ heating holder. The heating rate was 5 °C·min^–1^ and the holding time 30 min. The selected holding temperatures are 200, 250, 300, 325, 350, 375, and 400 °C, where high-angle annular dark-field (HAADF) micrographs were taken at the beginning, middle, and end of each holding period to minimize the effect of electron beam damage while monitoring the nanostructure evolution. The tilt angle of the lamella varied from −2.7° (before heating) to 11.8° (at 400 °C) to compensate for its bending with varying temperature without causing a significant contrast change.

### Mechanical Tests

After the X-ray measurements, the microsamples were transferred to a fresh Si substrate for mechanical tests. They were fixed on the Si substrate with Pt deposition via FIB (FEI Helios G3 UC SEM/FIB, Oregon, USA). The microcompression tests were carried out with a flat diamond punch (Synton-MDP, Nidau, CH) in an Agilent Nano Indenter G200 instrument (Agilent, Santa Clara, CA, USA). The samples were tested with loading–unloading cycles with an increasing maximum load. Based on previous microcompression tests,^5^ the maximum loads of the different cycles were selected to be 0.15, 0.2, 0.25, and 0.3 mN, with a final loading step that proceeded until the fracture of the samples. The loading rate was 10^–3^ mN·s^–1^. Three pillars and one supraparticle were tested (all after heat treatment and X-ray analysis). The fracture loads were 0.56, 0.67, and 0.18 mN for the pillars (with the last pillar loaded at the higher rate of 10^2^ mN·s^–1^), and 0.32 mN for the supraparticle, corresponding to 502, 722, 250, and 300 MPa, respectively. Note that for the supraparticle, this is a representative equivalent compressive strength, calculated as the applied load divided by the sphere’s equatorial cross-section, since a sphere under this loading condition experiences a distribution of tensile and compressive stresses at different domain areas. A more detailed analysis of the overall mechanical behavior of nanocomposite SPs has been conducted elsewhere.^73^

### Molecular Mechanics Simulations

The magnetite nanoparticle (NP), 4 nm in diameter, was generated via NanoCrystal^100^ with the force field parameters taken from our previous work.^101^ Undercoordinated Fe ions were hydroxylated. To ensure charge neutrality of the bare magnetite NP, Fe^2+^ and Fe^3+^ ions were randomly distributed by employing the charge neutrality equation introduced in our previous work. Fe^2+^ and Fe^3+^ oxidation states were then equilibrated using the oxidation state swap method.^101^

Subsequently, the NPs were functionalized with oleic acid molecules, with each functionalized magnetite NP containing approximately 17,000 atoms. An in-depth study detailing the application of this approach to the adsorption of organic molecules on the magnetite surface has previously been performed.^102^ This approach ensures that the oxidation states adapt to the surrounding electrostatic environment while maintaining compatibility with common biomolecular force fields. The hydroxylated nanoparticle was equilibrated using a hybrid Monte Carlo/Molecular Dynamics (MC/MD) method.^101^ The NP was subsequently functionalized with OLEC molecules.^103^ OLEC was described using GAFF^104^ parameters and RESP^105^ charges. The resulting functionalized NP was further used as a building block for the SCNCs to build and equilibrate an *fcc* SCNC. The *fcc* and *hcp* crystal structures have stacking order “ABC” and “AB” sequences, respectively. From the equilibrated *fcc* SCNC, an “ABCABCABABAB” system was created. All simulations were performed with LAMMPS.^106^ More details on the specific simulation protocols and force field details are provided in the SI section 5. Besides an optical inspection of the simulated structures, Steinhardt bond order parameters^86^ were used to determine the local supercrystalline structure. These parameters take into account the local orientational order by using spherical harmonics. Here, they are calculated employing the “compute orient-order/atom command” of LAMMPS for the nearest-neighbor shell of the NPs using their center of mass for the analysis. Particularly, the *q*_4_ and *q*_6_ Steinhardt bond order parameters were employed in this study, since they are highly effective for distinguishing between *fcc* and *hcp* local structures. For perfect lattices, fcc corresponds to *q*_4_ = 0.19 and *q*_6_ = 0.575, while hcp corresponds to *q*_4_ = 0.097 and *q*_6_ = 0.484. For nonideal systems, the values often deviate from the perfect ones and are found in a broader range; particularly for hcp, *q*_4_ is often between 0.05 and 0.15, while the others remain closer to the ideal values.^107,108^

## Supplementary Material



## Data Availability

Data available on request from the authors.

## Notes

Micropillars and supraparticles prepared for X-ray analysis (Section S1); microcompression tests (Section S2); AXCCA optimization of the unit cell parameters of Pillar and SP samples (Section S3); AXCCA of the anti-Mackay supraparticle (Section S4); all-atom simulations (Section S5); in situ heat treatment in the TEM (Section S6), including Figures S1–S15 (PDF)

## ▷

Institute of Applied Physics, University of Tübingen, Auf der Morgenstelle 10, 72076 Tübingen, Germany

## ▼

School of Earth & Planetary Sciences, National Institute of Science Education and Research, Jatani, 752050 Khurda, India; Homi Bhabha National Institute, Training School Complex, Anushaktinagar, 400094 Mumbai, India

## ⟠

Constructor University, Campus Ring 1, D-28759 Bremen, Germany
