# Supplementary material for: Defect Migration in Supercrystalline Nanocomposites
Source: ACS Nano. 2025 Dec 17;19(51):42881–96. doi: 10.1021/acsnano.5c16138 (PMC12756920; doi:10.1021/acsnano.5c16138)
Supplement: Supplementary file 1 [file nn5c16138_si_001.pdf]

Supplementary Information
for

Defect migration in supercrystalline nanocomposites

Dmitry Lapkin[1,*,i], Cong Yan[2], Emre Gürsoy[3], Hadas Sternlicht[4], Alexander Plunkett[5], Büsra Bor[5], Young Yong Kim[1], Dameli Assalauova[1,iii], Fabian Westermeier[1], Michael Sprung[1], Tobias Krekeler[6], Surya S. Rout[6,ii], Martin Ritter[6], Satishkumar Kulkarni[7], Thomas F. Keller[7,8], Gerold A. Schneider[5], Gregor B. Vonbun-Feldbauer[3,9], Robert H. Meissner[3,9], Andreas Stierle[7,8], Ivan A. Vartanyants[1], Diletta Giuntini[2,5,*]

[1] Deutsches Elektronen-Synchrotron DESY, Hamburg, 22607, Germany

[2] Department of Mechanical Engineering, Eindhoven University of Technology, Eindhoven, 5612 AP, Netherlands

[3] Institute for Interface Physics and Engineering, Hamburg University of Technology, Hamburg, 21073, Germany

[4] Department of Materials Science and Engineering, The Pennsylvania State University, University Park, 16802, PA, USA

[5] Institute of Advanced Ceramics, Hamburg University of Technology, Hamburg, 21073, Germany

[6] Electron Microscopy Unit, Hamburg University of Technology, Hamburg, 21073, Germany

[7] Centre for X-ray and Nano Science, Deutsches Elektronen-Synchrotron DESY, Hamburg, 22607, Germany

[8] Department of Physics, University of Hamburg, Hamburg, 22607, Germany

[9] Institute of Surface Science, Helmholtz-Zentrum Hereon, Geesthacht, 21502, Germany

[*] Corresponding authors

[i] Current address: Institute of Applied Physics, University of Tübingen, Auf der Morgenstelle 10, 72076 Tübingen, Germany

[ii] Current address: School of Earth & Planetary Sciences, National Institute of Science Education and Research, Jatani, 752050 Khurda, India; Homi Bhabha National Institute, Training School Complex, Anushaktinagar, 400094 Mumbai, India

[iii] Current address: Constructor University, Campus Ring 1, D-28759 Bremen, Germany

1. Micropillars and supraparticles prepared for X-ray analysis

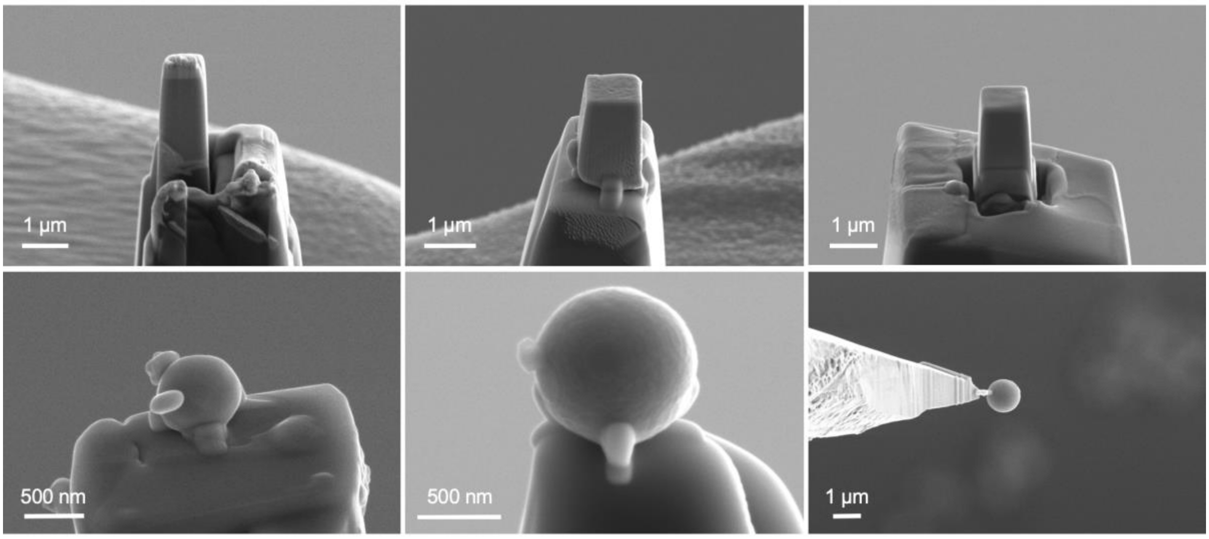

Fig. S1: Secondary electron images of micropillars ("Pillars") from bulk samples mounted on top of a pin suitable for the 3D X-ray analysis (top row) and supraparticles ("SPs") during and after the transfer via the micromanipulator onto the pin inside the dual beam focused ion beam instrument.

2. Microcompression tests

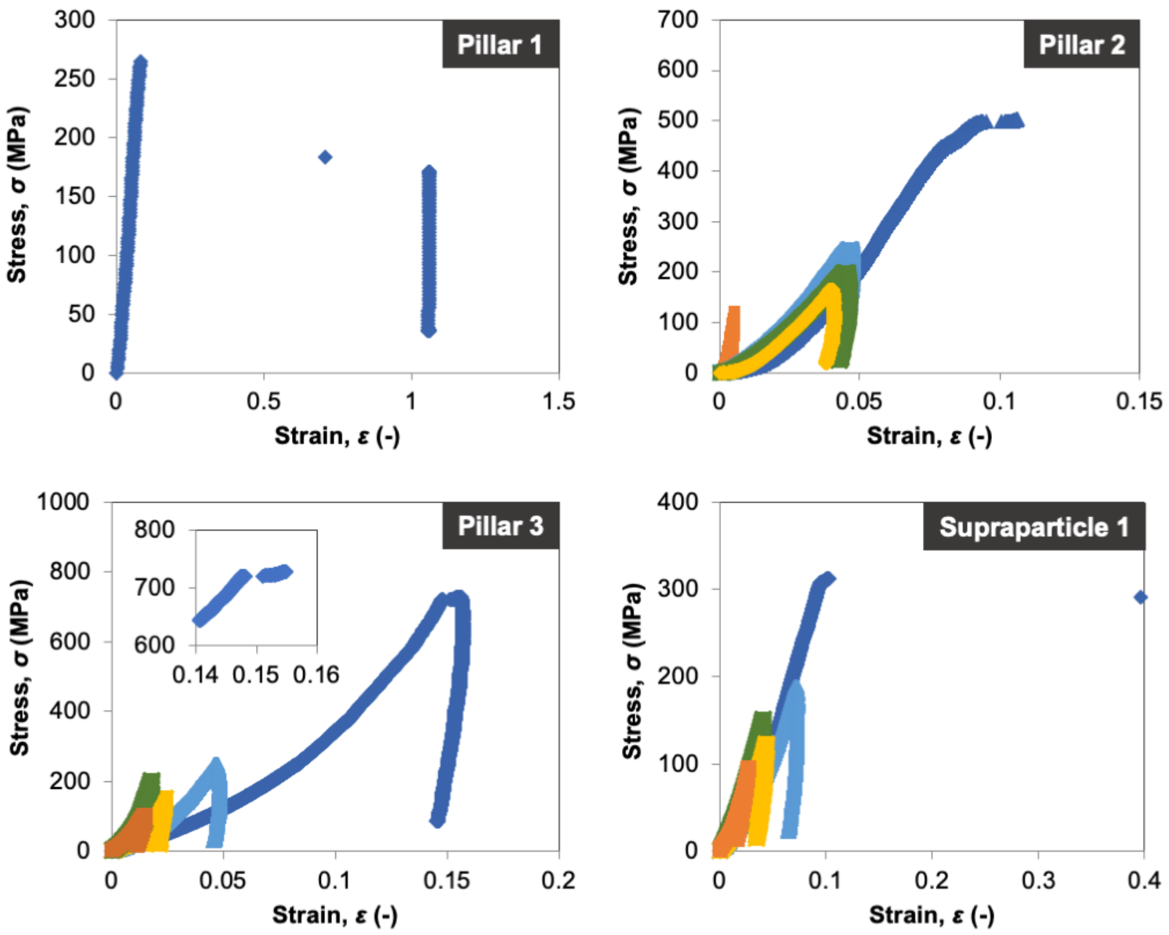

Fig. S2: Microcompression stress-strain curves for Pillars and SP, all in heat-treated (crosslinked) state. The other two SPs were damaged during sample transfer after the AXCCA analysis and could thus not be tested. The tests were conducted by loading and unloading each sample in compression in steps, until fracture. The colours indicate different test cycles, the dark blue colour indicates the cycle of final fracture.

3. AXCCA optimization of the unit cell parameters of Pillar and SP samples

The unit cell parameters were optimized as described in the Methods section in the main text.[1] Given the primitive unit cell parameters *a′*, *b′*, *c′* and *α′*, *β′*, *γ′*, we calculated the real and reciprocal basis vectors $\mathbf{a_1}$, $\mathbf{a_2}$, $\mathbf{a_3}$ and $\mathbf{b_1}$, $\mathbf{b_2}$, $\mathbf{b_3}$. Then, we calculated all expected peak positions (q_2,Δ) in the CCFs $C(q_1,q_2,\Delta)$ for the given unit cell parameters. The mean value ‹*C*› of the CCFs $C(q_1,q_2,\Delta)$ at the expected peak positions (q_2,Δ) was used as a metric. We found a global maximum of the metric and then tuned the unit cell parameters in a small range around the global maximum. The dependence of the ‹*C*› value on the parameters is shown in, e.g., Fig. S3. The positions of the maximum are treated as the average unit cell parameters, the HWHM (half width at half maximum) are treated as the distribution width and given in Tables 1 and 2 in the main text. One should note that the width of the peak is defined not only by the microstrain (the unit cell parameters distribution in the sample), but also by the sample size. Therefore, the obtained values give the upper estimations for the parameter distribution widths.

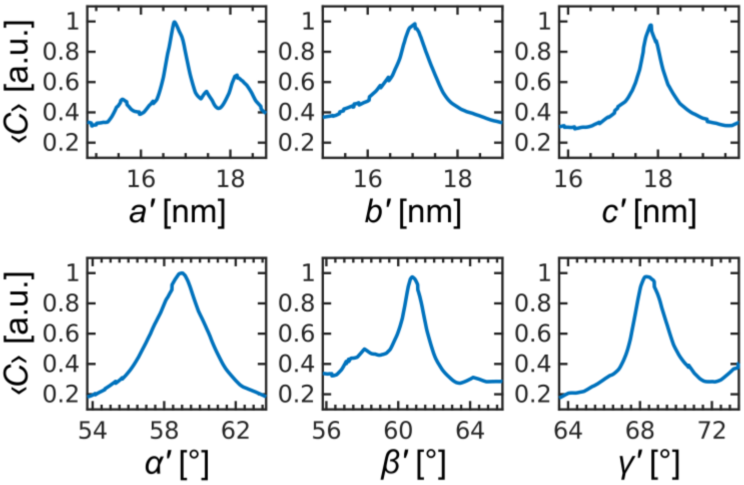

Fig. S3. Angular X-ray Cross-Correlation Analysis (AXCCA) of Pillar 1. Mean correlation values at the peak positions in the CCF map for Pillar 1 (shown in Fig. 2e in the main text) for a triclinic structure with the unit parameters *a*, *b*, *c*, *α*, *β*, *γ*. Each parameter is tuned separately, when all other are set to the ones giving the maximum value of the correlation.

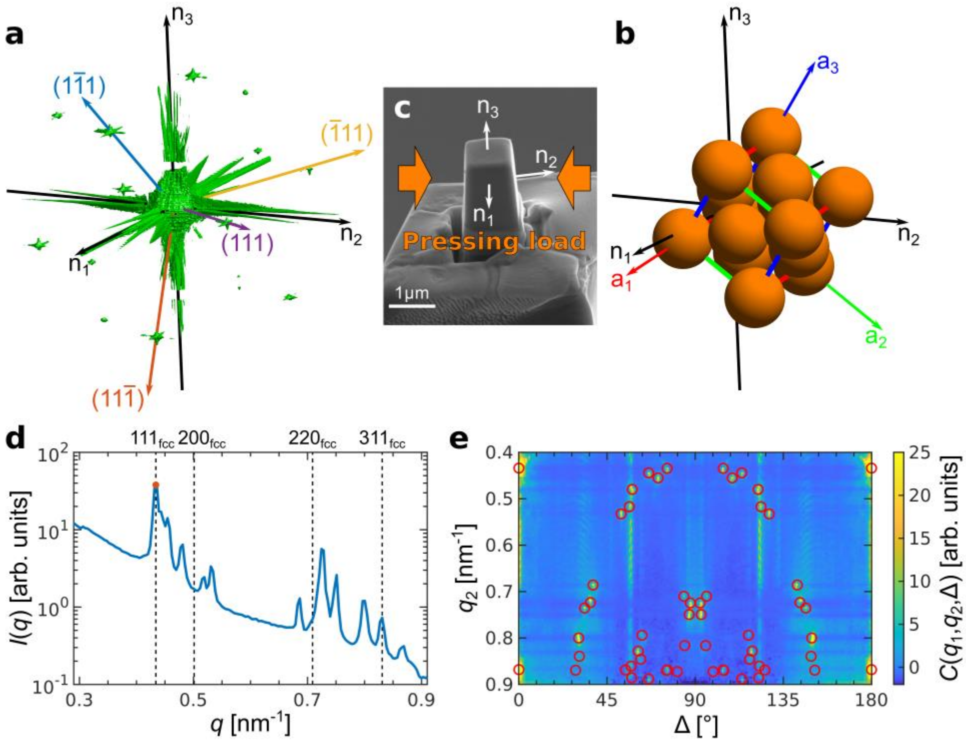

Fig. S4. Angular X-ray Cross-Correlation Analysis (AXCCA) of Pillar 2. a An isosurface of the measured scattered intensity distribution in 3D reciprocal space for Pillar 2. Four $(111)_{fcc}$ directions of the reciprocal lattice of a distorted *fcc* lattice are indicated by coloured arrows as well as the normal vectors $\mathbf{n_1}$, $\mathbf{n_2}$ and $\mathbf{n_3}$ to the pillar walls deduced from the intensity "spikes" orientation. **b** Orientation of a distorted *fcc* unit cell with respect to the pillar walls in real space. **c** An SEM image of the same pillar with indicated directions $\mathbf{n_1}$, $\mathbf{n_2}$ and $\mathbf{n_3}$. The uniaxial stress direction applied during the sample preparation is also indicated. **d** Azimuthally averaged intensity profile of the 3D scattered intensity of Pillar 2, with the red point indicating q_1 = 0.433 nm^{-1}, used for the calculation of the cross-correlation functions (CCFs). The peak positions of an ideal *fcc* structure are indicated with vertical dashed lines **e** CCFs $C(q_1,q_2,\Delta)$, calculated for q_1 (indicated in **d**) and q_2 in the range of 0.4 – 0.9 nm^{-1}, stacked along the vertical axis q_2, with the peak positions for the optimized unit cell parameters marked by red circles.

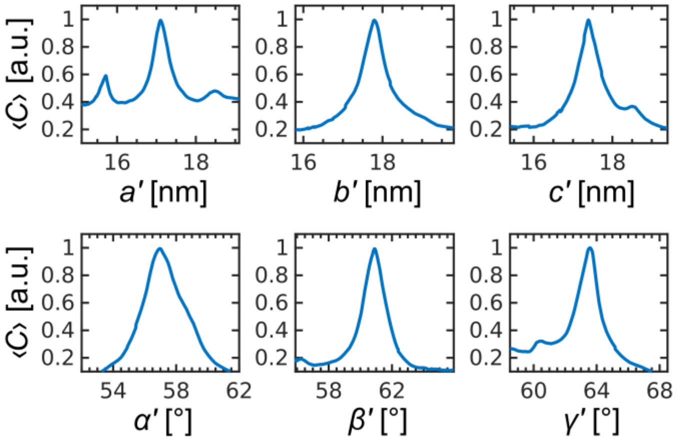

Fig. S5. Angular X-ray Cross-Correlation Analysis (AXCCA) of Pillar 2. Mean correlation values at the peak positions in the CCF map for Pillar 2 (shown in Fig. S4e) for a triclinic structure with the unit parameters *a*, *b*, *c*, *α*, *β*, *γ*. Each parameter is tuned separately, when all other are set to the ones giving the maximum value of the correlation.

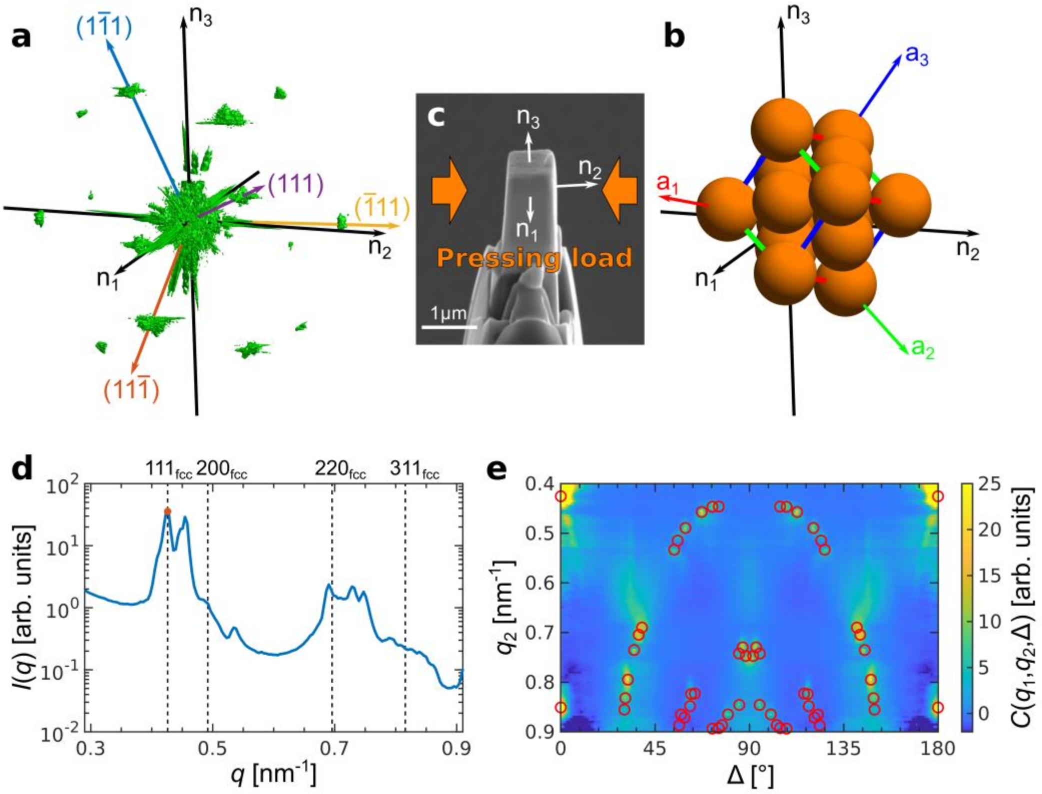

Fig. S6. Angular X-ray Cross-Correlation Analysis (AXCCA) of Pillar 3 before HT. a An isosurface of the measured scattered intensity distribution in 3D reciprocal space for Pillar 3 before HT. Four $(111)_{fcc}$ directions of the reciprocal lattice of a distorted *fcc* lattice are indicated by coloured arrows as well as the normal vectors $\mathbf{n_1}$, $\mathbf{n_2}$ and $\mathbf{n_3}$ to the pillar walls deduced from the intensity "spikes" orientation. **b** Orientation of a distorted *fcc* unit cell with respect to the pillar walls in real space. **c** An SEM image of the same pillar with indicated directions $\mathbf{n_1}$, $\mathbf{n_2}$ and $\mathbf{n_3}$. The uniaxial stress direction applied during the sample preparation is also indicated. **d** Azimuthally averaged intensity profile of the 3D scattered intensity of Pillar 3, with the red point indicating $q_1 = 0.426$ nm^{-1}, used for the calculation of the cross-correlation functions (CCFs). Peak positions for an ideal *fcc* structure are indicated with vertical dashed lines. **e** CCFs $C(q_1,q_2,\Delta)$, calculated for q_1 (indicated in **d**) and q_2 in the range of 0.4 – 0.9 nm^{-1}, stacked along the vertical axis q_2, with the peak positions for the optimized unit cell parameters marked by red circles.

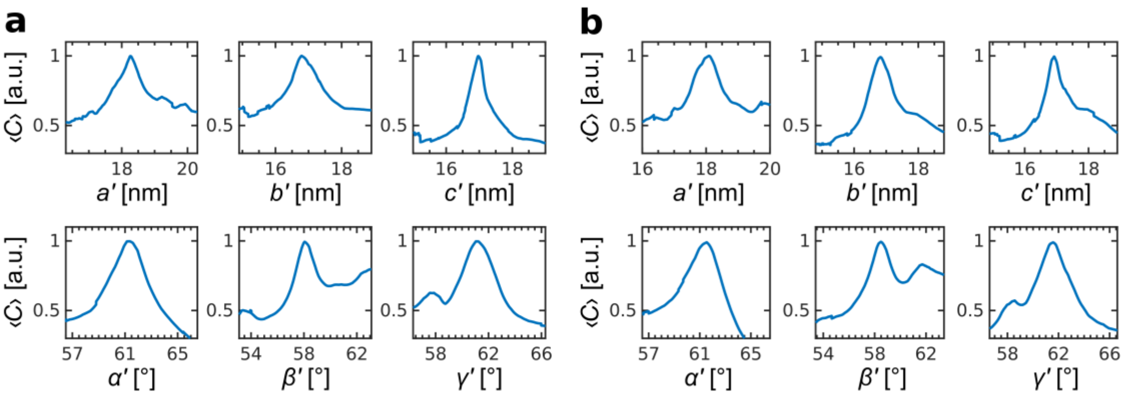

Fig. S7. Angular X-ray Cross-Correlation Analysis (AXCCA) of Pillar 3 before (a) and after (b) heat treatment. Mean correlation values at the peak positions in the CCF map for Pillar 3 (shown in Fig. S6e) for a triclinic structure with the unit parameters *a*, *b*, *c*, *α*, *β*, *γ*. Each parameter is tuned separately, when all other are set to the ones giving the maximum value of the correlation.

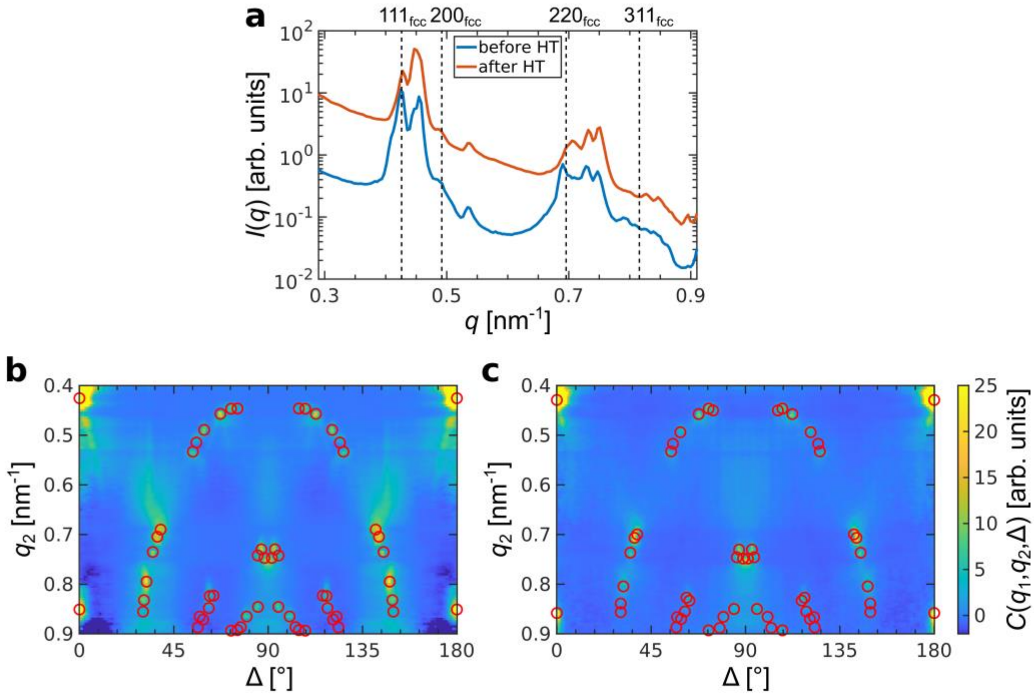

Fig. S8: Angular X-ray Cross-Correlation Analysis (AXCCA) of Pillar 3 before and after heat treatment. a Azimuthally averaged intensity profiles of the 3D scattered intensities of Pillar 3 before (blue line) and after (red line) heat treatment. The profiles are shifted vertically for clarity. Peak positions of an ideal *fcc* structure are indicated with vertical dashed lines. **b, c** CCFs $C(q_1,q_2,\Delta)$ calculated for q_1 corresponding to the 111_{fcc} Bragg peak and q_2 in the range of 0.4 – 0.9 nm^{-1} before (**b**) and after (**c**) heat treatment. The CCFs are shown as a heat map stacked along vertical axis q_2.

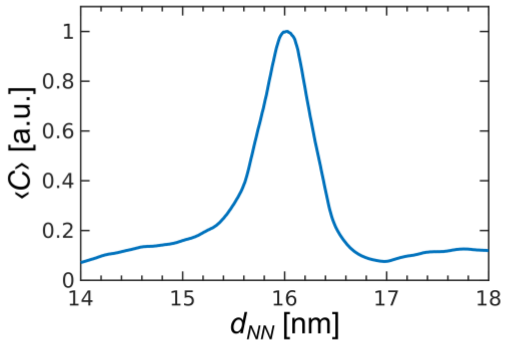

Fig. S9. Angular X-ray Cross-Correlation Analysis (AXCCA) of SP 1. Mean correlation values at the peak and arc positions in the CCF map for SP 1 (shown in Fig. 3c in the main text) for an *fcc* structure as a function of the nearest neighbor distance d_{NN}.

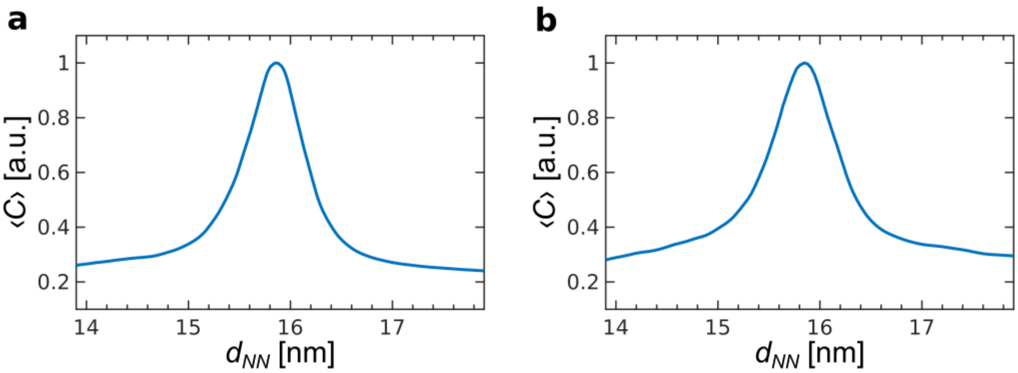

Fig. S10. Angular X-ray Cross-Correlation Analysis (AXCCA) of SP 3 before (a) and after (b) heat treatment. Mean correlation values at the peak and arc positions in the CCF map for SP 3 (shown in Figs. 4d,e in the main text) for an *fcc* structure as a function of the nearest neighbor distance d_{NN}.

4. AXCCA of the anti-Mackay supraparticle

The optimized particle positions of the anti-Mackay structure in a SP of the corresponding size were obtained based on previous works from FAU Erlangen.[2–4] The particle positions were used to calculate 2D X-ray scattering patterns for the same details as in the experiment (see the Methods section of the main text). The 2D pattern were simulated using the MOLTRANS software. The obtained 2D patterns were interpolated onto a 3D grid to obtain the entire 3D scattered intensity distribution. The CCFs $C(q_1,q_2,\Delta)$ were then calculated for q_1 corresponding to the 111_{fcc} Bragg peak and q_2 varied in the range of 0.4 – 0.9 nm^{-1}. The resulting CCFs of the simulated intensity distribution is shown in Fig. S11b for comparison with the experimental CCFs shown in Fig. S11a. The regions of the 111_{fcc} and 200_{fcc} Bragg peaks ($q \approx 0.475$ nm^{-1} and $q \approx 0.775$ nm^{-1}, respectively) are very similar and contain the same characteristic peaks for the anti-Mackay structure, which are distinctive from those of a simple *fcc* structure. On the other hand, the CCFs calculated for the simulated intensity distribution contain many additional peaks in the q-range between the Bragg peaks (q = 0.5 – 0.75 nm^{-1}) because the simulation did not include any noise hiding the correlations in the experimental data.

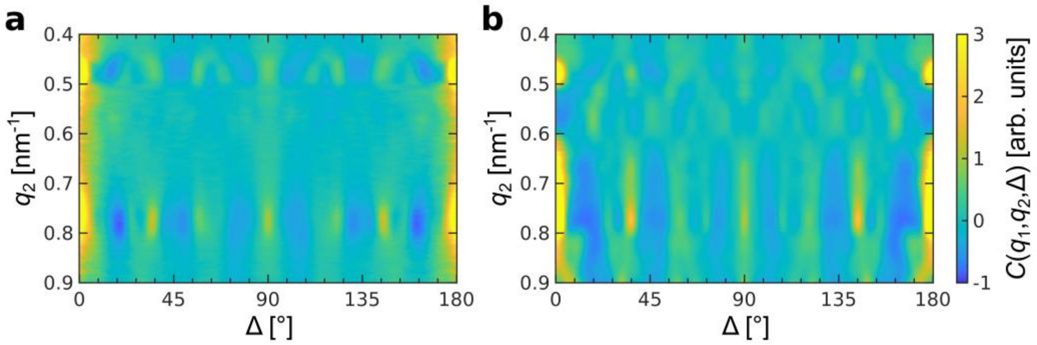

Fig. S11. Angular X-ray Cross-Correlation Analysis (AXCCA) of SP 2 with anti-Mackay structure. CCFs $C(q_1,q_2,\Delta)$ calculated for q_1 corresponding to the 111_{fcc} Bragg peak and q_2 in the range of 0.4 – 0.9 nm^{-1} for experimental (**a**) and simulated (**b**) 3D scattered intensity distributions. The CCFs are shown as a heat map stacked along the vertical axis q_2.

5. All-atom simulations

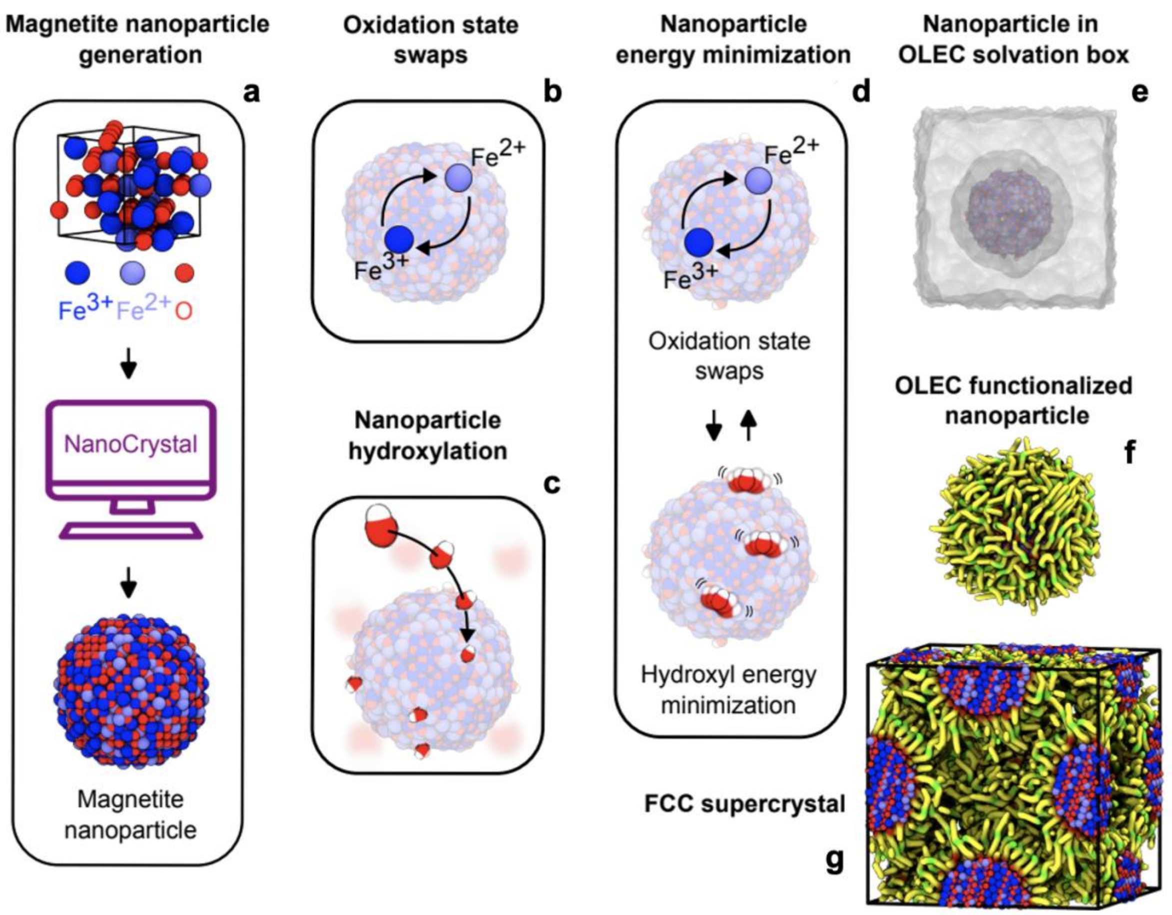

Fig. S12. Multiscale workflow for generating all-atom OLEC functionalized magnetite SCNCs. a Generation of a magnetite NP, **b** oxidation state swaps and **c** hydroxylation of NP; **d** Minimization of potential energy; **e** Functionalization of NP in OLEC solvation box; **f** OLEC functionalized NP building block to construct the SCNC (**g**).

Magnetite nanoparticle generation

A spherical magnetite nanoparticle (NP) with a diameter of 4 nm based on an $Fd\bar{3}m$ unit cell (see Fig. S12a) was generated using NanoCrystal[5]. This is a web-based tool that generates NP models using their crystal structure, desired size, preferred growth planes and energies. The coordination polyhedron option was not used. After NP generation, the partial atomic charges of Fe ions were randomly distributed to ensure charge neutrality using the charge neutrality equation for non-stoichiometric magnetite particles or surfaces.[6] Forcefield parameters describing magnetite NP were taken from previous work.[7]

NP oxidation state equilibration

Oxidation states of magnetite NPs were distributed using oxidation state swaps[6] (see Fig. S12b). The oxidation state swap method is an atomistic simulation method that was developed specifically for modelling magnetite structures while ensuring compatibility to common biomolecular force fields. It is based on exchanging oxidation states of Fe ions using a Monte Carlo (MC) approach, with or without the combination of Molecular Dynamics (MD). To minimize the oxidation states of the NPs inside a 150 × 150 × 150 $Å^3$ simulation box with non-periodic boundary conditions MC swaps were performed using LAMMPS[8]. For pairwise interactions, instead of using a long-range solver, a relatively large cut-off of 45 Å was used. This cut-off ensured that the interactions between magnetite ions on opposite sides of the NP were taken into account. $n_{\mathrm{swaps}} \approx (n_{\mathrm{Fe}})^2$ swaps were performed at T^{MC} = 300 K temperature.

NP hydroxylation and energy minimization

With the setting "coordination polyhedral" turned off, the spherical NP produced using NanoCrystal[5] is not stoichiometric. Particularly, the generated NP contained eleven oxygen atoms less than for the ideal magnetite stoichiometry. To ensure magnetite stoichiometry, the eleven least coordinated Fe ions on the surface were hydroxylated (Fig. S12c). Energy minimization and oxidation state swaps were performed on the hydroxylated NP in cyclic order. During the energy minimization step, magnetite ions were kept frozen. After each minimization step, oxidation state swaps were applied to allow the Fe ions to adapt to the updated hydroxyl geometry (Fig. S12d). At each oxidation state swap step, approximately $n_{\mathrm{swaps}} \approx (n_{\mathrm{Fe}})^2$ were performed at T^{MC} = 300 K. In total, ten cycles of energy minimization and oxidation state swaps were performed. For pairwise interactions non-periodic boundary conditions were used with a relatively large cut-off of 55 Å to ensure that interactions between hydroxides on opposite sides of the NP were accounted for. Following potential energy minimization, the hydroxylated NP was equilibrated using hybrid Monte Carlo/Molecular Dynamics (MC/MD)[6] for 1 ns with a timestep of 0.5 fs. At each MD step, one MC swap was

attempted, and both the T^{MD} and T^{MC} were held at 300 K. Pairwise interactions of bonded atoms in hydroxyl groups were scaled to zero.

Oleic acid solvation box generation and equilibration

Oleic acid (OLEC) molecules, i.e. C18H34O2, were modelled using GAFF[9] parameters and RESP charges.[10] A 150 × 150 × 150 Å^3 simulation box was randomly filled with 2000 OLEC molecules. The OLEC box was equilibrated in the *NpT* ensemble starting from 300 K temperature and 100 atm pressure and gradually lowering the pressure until 1 atm over a period of 1 ns while keeping the temperature constant. Running the simulation at this relatively high pressure avoids potential OLEC aggregation (bubble formation), while allowing OLEC molecules to reorient. After the initial equilibration, a follow-up *NpT* simulation was performed for 1 ns at 300 K and 1 atm pressure to equilibrate the liquid OLEC box. As a result, an OLEC density of 0.88 g·cm^{-3} was obtained which agrees well with the experimental OLEC density at room temperature of 0.89 g·cm^{-3}. For pairwise interactions, a PPPM[11] solver with a precision of 10^{-6} and a real-space cutoff of 12 Å was used. For bonded atoms, pairwise interactions associated with 1-2, 1-3 and 1-4 terms were scaled to fit GAFF.

Magnetite NP functionalization

A cavity approximately as large as the hydroxylated NP was carved out of the liquid OLEC box. The hydroxylated NP was then placed in this cavity (see Fig. S12e). Initially the combined system was sampled at 500 K temperature for 10 ns using an *NVE* ensemble combined with a CSVR[12] thermostat, allowing OLEC molecules to reorient. Subsequently, the system was sampled via an *NpT* ensemble starting from 500 K and 100 atm pressure which was gradually reduced to a temperature of 300 K over 5 ns while the pressure was kept constant. This was followed by another *NpT* equilibration at 300 K and 100 atm, during which the pressure was gradually lowered to 1 atm over 5 ns while the temperature was kept constant. For pairwise interactions, a PPPM[11] solver with a precision of 10^{-6} and a real-space cutoff of 12 Å and

above mentioned 1-2, 1-3, 1-4 scaling factors of GAFF were used. To calculate the OLEC coverage, the number of carboxylic hydrogens within 6 Å of the NP surface was determined and divided by the NP's surface area (see Fig S13). While the first coordination shell of carboxylic hydrogens is within 2 Å from the magnetite surface, the larger distance of 6 Å was specifically chosen to also include OLEC molecules close to surface hydroxide groups.

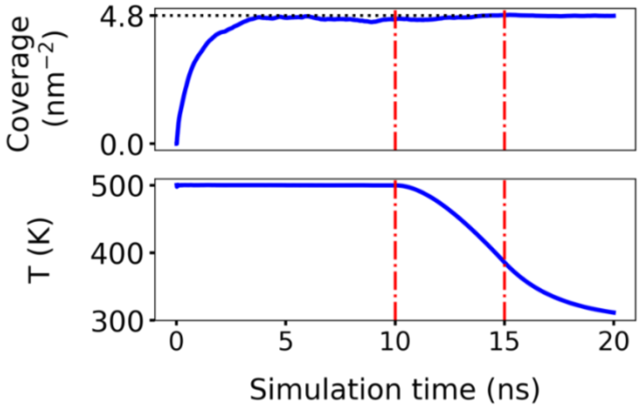

Fig. S13. Oleic acid coverage on the magnetite nanoparticle (NP). The upper figure shows the time-dependent evolution of the oleic acid coverage on the magnetite NP, calculated as the number of carboxylic hydrogens within 6 Å of the NP surface divided by NP surface area. The lower figure depicts the temperature during the molecular dynamics simulation.

SCNC generation and equilibration

The non-bonded OLEC molecules were removed from the system, resulting in an 16925-atom OLEC-functionalized magnetite NP (Fig. S12f), which was used as the building block to construct SCNCs (Fig. S12g). An *fcc* SCNC was generated using Moltemplate[13] and the [111] direction of the *fcc* structure was aligned to the z-axis of the simulation box corresponding to the close-packed direction. Subsequently, the structure was equilibrated for 10 ns using *NpT* ensemble at 1 atm pressure and 300 K temperature. To use a relatively large timestep of 1 fs, all bonds involving hydrogen were constrained to their original length using the SHAKE algorithm. Contrary to before, in order to speed up the much larger simulations, A PPPM[11] solver with a precision of 10^{-4} was used.

6. In situ heat treatment in the TEM

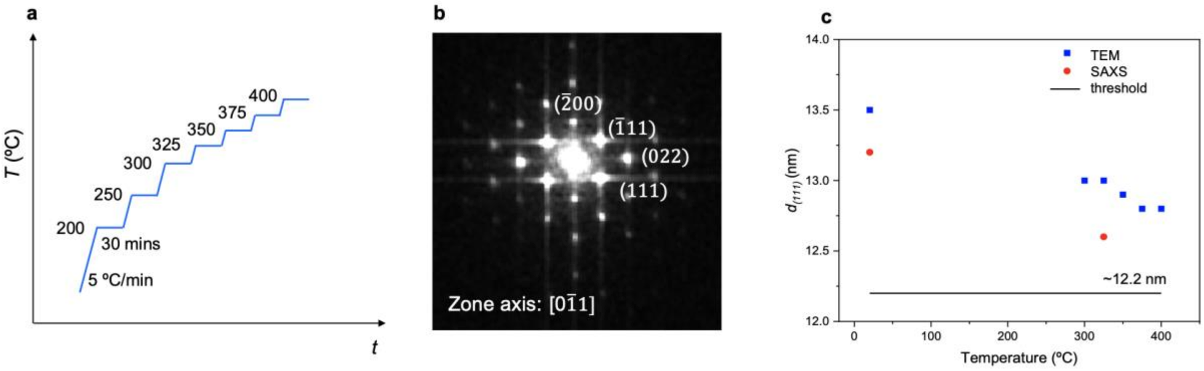

Fig. S14. In situ heat treatment in the TEM. a Temperature profile applied during the experiment. **b** Identification of supercrystalline planes reflections and indication of zone axis. **c** Changes in the distance between (111) planes with increasing heat treatment temperature. TEM values are estimated as distances between inverse FFT fringes, SAXS ones are global values obtained for the whole SCNC sample from which the lamella is extracted. Note that the TEM values only serve as indication here, since both abutting grains are not aligned in zone axis, but rather near it, and minor sample tilts, bending and potential beam damage to the organic material do not allow the interplanar distances to be determined accurately. The discrepancy between TEM and SAXS values is then attributed to these factors, and to the fact that SAXS provides a global average over the whole mm-scale sample. Even in the case of SAXS, however, the inter-NP distances remain above the 12.2 nm limit that corresponds to the onset of sintering (NPs in contact with each other).

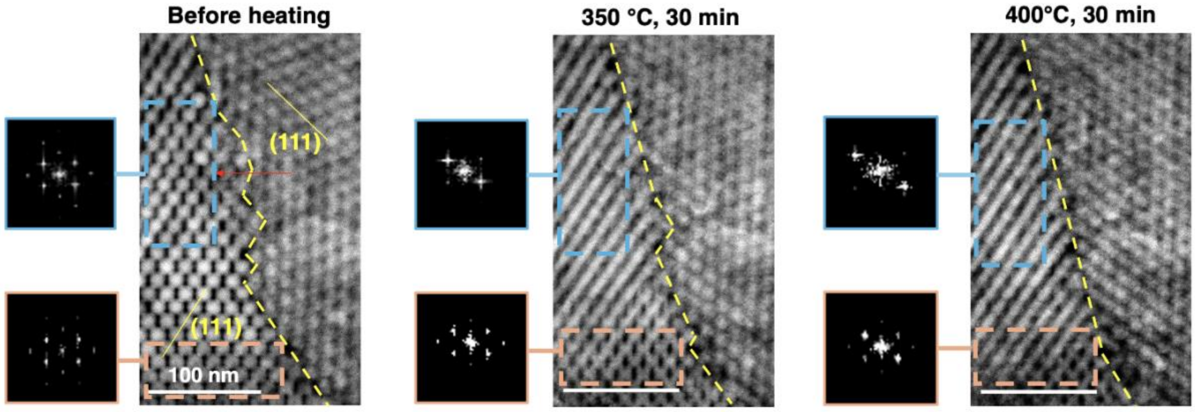

Fig. S15. Supercrystalline domains and boundary. Due to slight bending, tilt and organic removal in the lamella analysed in the TEM, the left SCNC domain might appear to transition into two domains with different orientations. An FFT analysis on two separate areas of the same domain confirms that its orientation remains uniform throughout the heat treatment.

References

1. Lapkin, D. *et al.* Angular X-ray cross-correlation analysis applied to the scattering data in 3D reciprocal space from a single crystal. *IUCrJ* **9**, 425–438 (2022).
2. Wang, J. *et al.* Free Energy Landscape of Colloidal Clusters in Spherical Confinement. *ACS Nano* **13**, 9005–9015 (2019).
3. Mbah, C. F. *et al.* Early-stage bifurcation of crystallization in a sphere. *Nature Communications 2023 14:1* **14**, 1–9 (2023).
4. Wang, J. *et al.* Magic number colloidal clusters as minimum free energy structures. *Nature Communications 2018 9:1* **9**, 1–10 (2018).
5. Chatzigoulas, A., Karathanou, K., Dellis, D. & Cournia, Z. Correction to "NanoCrystal: A Web-Based Crystallographic Tool for the Construction of Nanoparticles Based on Their Crystal Habit". *J Chem Inf Model* **59**, 1681 (2019).
6. Gürsoy, E., Vonbun-Feldbauer, G. B. & Meißner, R. H. Oxidation-State Dynamics and Emerging Patterns in Magnetite. *Journal of Physical Chemistry Letters* **14**, 6800–6807 (2023).
7. Konuk, M., Sellschopp, K., Vonbun-Feldbauer, G. B. & Meißner, R. H. Modeling Charge Redistribution at Magnetite Interfaces in Empirical Force Fields. *Journal of Physical Chemistry C* **125**, 4794–4805 (2021).
8. Thompson, A. P. *et al.* LAMMPS - a flexible simulation tool for particle-based materials modeling at the atomic, meso, and continuum scales. *Comput Phys Commun* **271**, 108171 (2022).
9. Wang, J., Wolf, R. M., Caldwell, J. W., Kollman, P. A. & Case, D. A. Development and testing of a general amber force field. *J Comput Chem* **25**, 1157–1174 (2004).
10. Creutzburg, M. *et al.* Adsorption of oleic acid on magnetite facets. *Communications Chemistry 2022 5:1* **5**, 1–9 (2022).
11. Hockney, R. W. & Eastwood, J. W. Computer Simulation Using Particles. *Computer Simulation Using Particles* (2021) doi:10.1201/9780367806934.
12. Bussi, G., Donadio, D. & Parrinello, M. Canonical sampling through velocity rescaling. *Journal of Chemical Physics* **126**, (2007).
13. Jewett, A. I. *et al.* Moltemplate: A Tool for Coarse-Grained Modeling of Complex Biological Matter and Soft Condensed Matter Physics. *J Mol Biol* **433**, 166841 (2021).
